# Automated workflow for target volume propagation in online adaptive magnetic resonance guided radiotherapy for lung lesions using foundation models

**DOI:** 10.1016/j.phro.2026.101077

**Published:** 2026-09-07

**Authors:** Denis Dudas, Tom Julius Blöcker, Miguel A. Palacios, Chengtao Wei, Chukwuka Eze, Stefanie Corradini, Claus Belka, Christopher Kurz, Guillaume Landry

**Affiliations:** a Department of Radiation Oncology, LMU University Hospital, LMU Munich, Munich, Germany; b Department of Radiation Oncology, Amsterdam UMC, VU University Medical Center, Amsterdam, the Netherlands; c Department of Radiation Oncology, Universitätsklinikum Erlangen, Friedrich-Alexander-Universität Erlangen-Nürnberg (FAU), Erlangen, Germany; d Bavarian Cancer Research Center (BZKF), Munich, Germany; e German Cancer Consortium (DKTK), Partner Site Munich, a Partnership Between DKFZ and LMU University Hospital Munich, Munich, Germany

**Keywords:** Adaptive radiotherapy, Foundation models, Contour propagation, Deep learning, Image guidance

## Abstract

**Background and purpose:**

This study presents a novel workflow for automated gross tumor volume (GTV) adaptation in online adaptive magnetic resonance imaging (MRI)-guided radiotherapy using promptable segmentation foundation models and evaluates its impact on the quality of adapted treatment plans.

**Materials and methods:**

Treatment planning GTV contours in lung cancer patients, treated at an MRI-Linac, were propagated to daily MRI using affine or deformable image registration. Subsequently, propagated contours were refined using MedSAM2 or nnInteractive foundation models. The prompting strategy was optimized using an internal validation dataset (16 patients with single GTV, 143 fractions), and its accuracy was evaluated on internal and external datasets (total of 39 patients with single GTV, 225 fractions). In the dose-volume histogram (DVH) evaluation, daily treatment plans were reoptimized to GTVs and planning target volumes (PTV) generated by the proposed workflow. The resulting DVHs were compared with the originals.

**Results:**

NnInteractive improved the segmentation accuracy significantly compared to affine- and TransMorph-based propagation methods. The Dice similarity coefficient improved by up to 15% compared to affine-based propagation, but only marginal improvements were observed compared to TM-based propagation. The DVH evaluation revealed no systematic degradation of plan quality. Most DVH metrics did not differ significantly from the original treatment plans. In 17 out of 21 cases, 100% of the clinically approved GTV volume remained covered by the prescribed dose after the reoptimization.

**Conclusion:**

The proposed workflow is feasible and enables automated daily GTV adaptation with improved segmentation accuracy and comparable quality of adapted treatment plans.

## Highlights

• Foundation models enable daily tumor contour adaptation on MRI-Linac.
• Two foundation models were evaluated: nnInteractive and MedSAM2.
• nnInteractive increased the Dice score up to ∼15% for affine propagation.
• nnInteractive increased the Dice score up to ∼5% for TransMorph propagation.
• Tumor coverage was maintained in 17 of 21 reoptimized treatment plans.

## Graphical abstract

**Figure:**
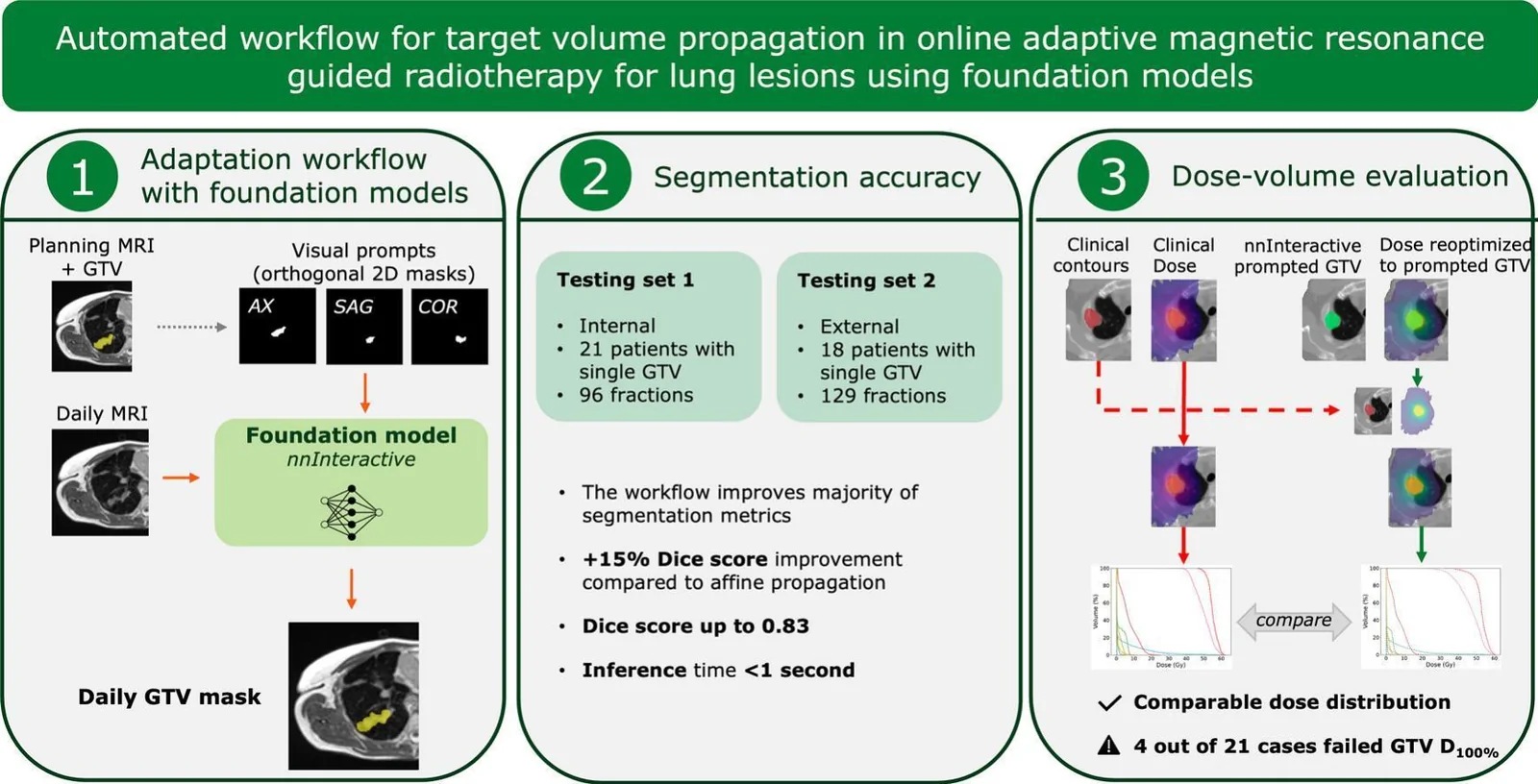

## 1 Introduction

The magnetic resonance imaging (MRI) - linear accelerator (linac) (MRI-Linac), enables online adaptive radiotherapy by combining MRI for daily treatment adaptation with a linac for treatment delivery. This platform is particularly useful in lung cancer radiotherapy, where the high contrast between the target volume and surrounding healthy tissue is ideal for daily adaptation [1], [2], [3]. A key and time-consuming step in the online adaptive workflow is the adaptation of target volume and organ-at-risk (OAR) contours [1], [4]. A previous study by Güngör et al. [5] reported a median contour adaptation time of approximately 45 min, from which 9 min were spent on tumor volume contour adaptation. More recent study by Westerhoff et al. [6] reported mean time required for full online adaptation of approximately 25 min.

The current state of the art for contour adaptation on MRI-Linacs in lung cancer is based on contour propagation using rigid/affine or deformable image registration (DIR) [7], [8], [9], [10]. Planning contours are propagated to the daily MRI and then reviewed and manually edited by a radiation oncologist before approval, which drives the added workload and time. Multiple earlier studies have addressed online contour adaptation using deep learning, via auto-segmentation [11] or DIR [12], [13]. A promising DIR architecture is the transformer-based TransMorph (TM) model [14]; Wei et al. [15] applied it to contour propagation in MRI-guided lung radiotherapy and reported superior performance over conventional baselines such as B-spline DIR and nnU-Net [16]. Despite substantial progress in deep-learning auto-segmentation [17], [18], [19], [20], target volumes remain more heterogeneous than OARs in location, shape and appearance, which limits model generalizability [21]. A promising direction is segmentation foundation models, which enable single-shot segmentation and generalize across imaging modalities, anatomical sites, and target structures [22], [23], [24].

This feasibility study presents a workflow for automatic target-volume adaptation in lung lesions using segmentation foundation models, prompted by clinically approved planning contours to guide segmentation on daily MR images. The accuracy of this workflow and its impact on the plan quality of the adapted daily treatment plans were evaluated.

## 2 Material and methods

### 2.1 Study datasets

The study included stage I–II lung cancer and lung oligometastasis patients treated with stereotactic body radiation therapy (SBRT) on 0.35 T MRIdian MR-Linac systems (ViewRay Systems Inc., USA) at two institutions, using the same workflow and MRI sequences described by Finazzi et al. [4]. The study followed the Declaration of Helsinki; ethics approval and informed consent were obtained at both institutions (LMU Klinikum, approval 23–1043; Amsterdam University Medical Centers [AUMC], approval 2018.602).

The available data were divided into three datasets, all with single gross tumor volume (GTV) patients: a validation dataset (internal, LMU Klinikum, 16 patients, 143 fractions), testing dataset 1 (internal, LMU Klinikum, 21 patients, 96 fractions) and testing dataset 2 (external, AUMC, 18 patients, 129 fractions). The validation dataset served to optimize the prompting strategy, the testing datasets for independent evaluation of the workflow. All datasets included planning and daily images with clinically approved (adapted) contours; prescribed fractionation varied (3, 5, 8 and 10 fractions; see supplementary material, Table S.1). The internal datasets additionally included complete treatment plans with optimization objectives and constraints, required for the dose-volume histogram (DVH) evaluation (Section 2.4). External plans and optimization objectives were unavailable.

### 2.2 GTV mask propagation

All images were center-padded or cropped to 320 × 224 × 160 voxels (spacing 1.5 × 1.5 × 3 mm${\;}^{3}$, LR×AP×SI) and rescaled to [0,1] with 99.5th-percentile clipping. The planning GTV was propagated to the daily MRI by two baseline methods: affine registration, and DIR using the TM model. Affine registration was performed using the open-source SimpleElastix framework with default configuration, while DIR was carried out using the TM model published by Wei et al. [15]. Two propagation methods and two foundation models were compared, each pair representing two clinically relevant alternatives: affine registration is the fast baseline available on the MRIdian system and TM a state-of-the-art deep-learning DIR method [15], while MedSAM2 is a medical-domain–tuned, video-style propagation model and nnInteractive a native 3D nnU-Net–based model. This allowed assessment of whether foundation-model refinement is beneficial irrespective of the starting contour.

**Fig. 1:**
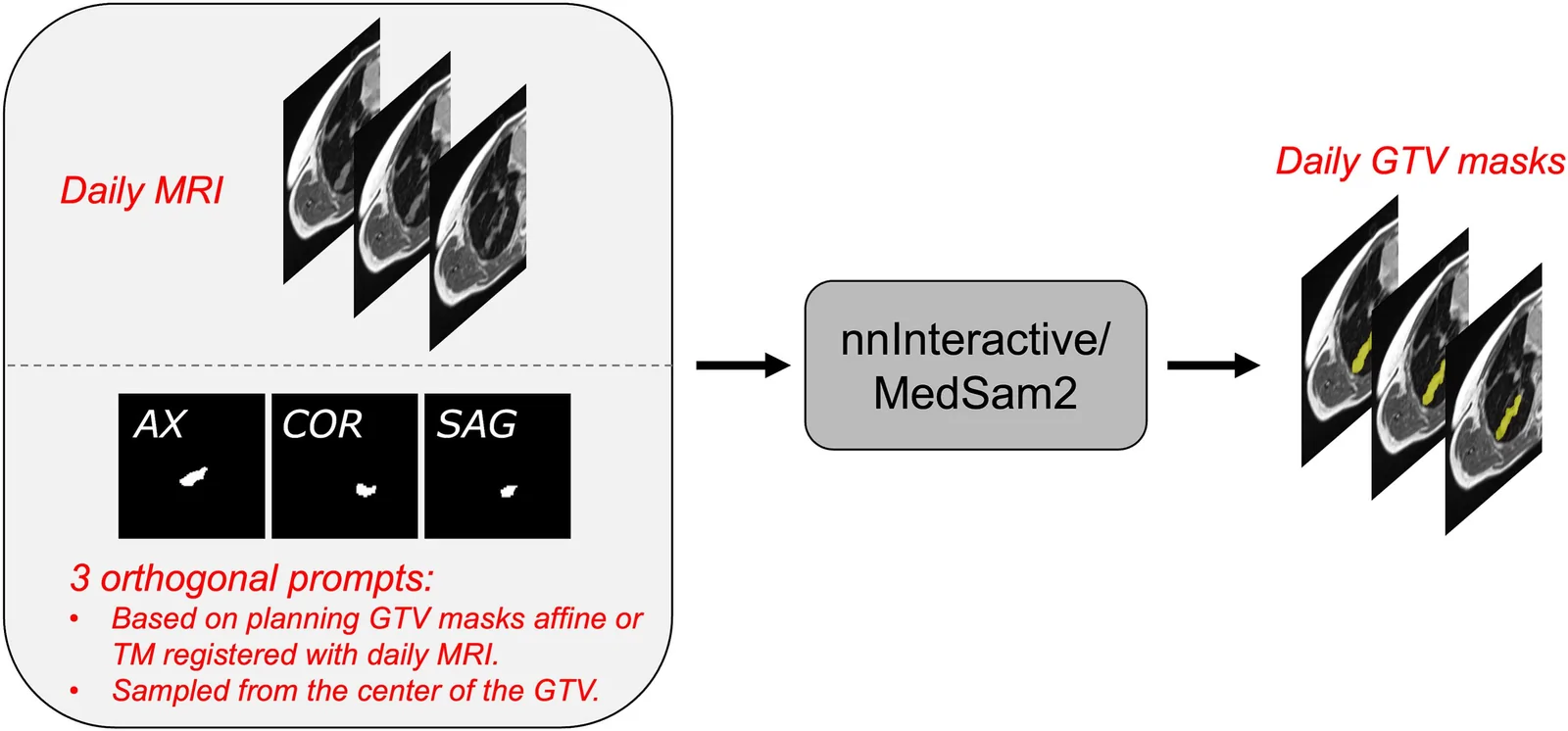
Schematic overview of the proposed GTV adaptation workflow using foundation models. The input consists of daily MRI and 3 orthogonal prompts (masks in axial, sagittal and coronal orientation), derived from the planning GTV mask. The planning MRI is first registered to the daily MRI using either affine or TM registration. At the output, there is the daily adapted GTV mask.

The propagated masks (affine- or TM-based) were then refined using promptable segmentation foundation models, which take the daily MRI and a set of visual prompts as input and output the daily GTV adaptation, GTV^AI^ (Fig. 1). The prompting strategy was optimized on the validation dataset: three automatically generated orthogonal masks with one-voxel dilation, intersecting at the GTV center, performed best, with no other strategy giving substantial improvement. This is consistent with Blöcker et al. [25], who reported the same strategy as superior to alternatives.

### 2.3 Promptable foundation models and statistical analysis

In this study, two publicly available segmentation foundation models were investigated - MedSAM2 and nnInteractive [22], [23]. Even though their implementations differ, a common feature is the use of visual prompts, which allow the user to guide the model in segmentation of specific objects. Supported prompt types include negative and positive point prompts, bounding boxes, masks or scribbles. Both models were compared on the validation dataset, and only the better-performing model (nnInteractive; see Results) was carried forward into the testing and DVH evaluation phases.

MedSAM2 [26] is a medical-domain–tuned version of the SAM2 video-segmentation model (SAM2.1‑tiny), fine-tuned on a large dataset of 3D medical images. As SAM2 is designed for video, 3D segmentation is performed by prompting an initial slice and propagating the mask bidirectionally to adjacent slices. Here, the initial slice was the central slice of the planning data registered to the daily MRI by affine- or TM-based registration, i.e. the slice through the center of the tumor as defined by the registered planning contour and therefore a tumor-centered anatomical reference. It was placed before the corresponding slices of the daily imaging data and prompted with the registered planning contour, after which the GTV mask was propagated bidirectionally; this directly simulated temporal tracking of the GTV over daily MRI data. The procedure was repeated in all three orthogonal orientations, and the final GTV mask was the intersection of the three resulting masks. The second model, nnInteractive [22], is a foundation model for 3D medical segmentation based on the nnU-Net architecture, with visual prompts as additional input channels; it was trained from scratch on more than 700,000 object masks.

Both models required approximately 300 ms per prompt for a 2D mask (NVIDIA RTX A6000 GPU, NVIDIA Corporation, USA). The total inference time for the proposed workflow, i.e. prompting with three orthogonal prompts, was approximately 1 s. Baseline and foundation model–refined masks were compared on the validation and both testing datasets using Dice Score (DSC), 50th/95th-percentile Hausdorff Distance (HD50/HD95), Surface Dice Score (sDSC; one-voxel tolerance, 1.5 × 1.5 × 3 mm), Precision, and Recall, with significance assessed by the Wilcoxon signed-rank test.

### 2.4 DVH evaluation of the workflow

**Fig. 2:**
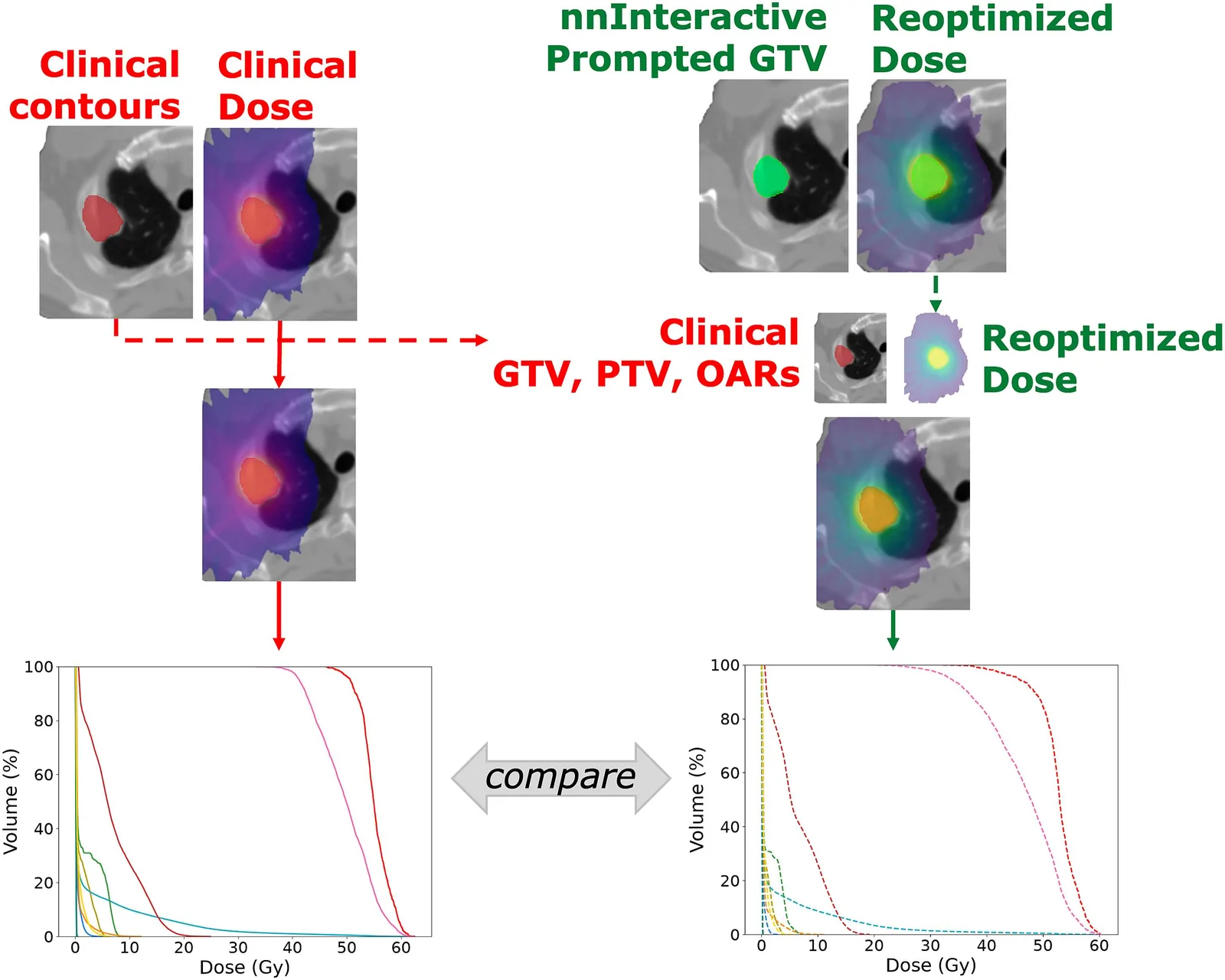
Schematic overview of the DVH evaluation. DVHs corresponding to the original clinically approved adapted plan were compared with the reoptimized DVHs. The adapted plan was reoptimized to the GTV^AI^ from the proposed workflow using the same optimization constraints and objectives. The reoptimized dose distribution was subsequently applied to original clinically approved GTV, PTV and OARs which resulted in another set of DVHs that could be compared to the original ones.

This part of the study evaluated the impact of the workflow on the plan quality of adapted plans, a step earlier identified as crucial for clinical implementation of automatic contours in adaptive radiotherapy [27]. The aim was to assess whether plans optimized to GTV^AI^ and the corresponding planning target volume (PTV)^AI^ yield similar DVH metrics when evaluated on the original clinically approved GTV, PTV, and OAR contours (a fraction-level comparison). Based on the testing results, TM + nnInteractive refinement was used. The analysis was performed on the internal testing dataset (21 patients with single lesions). One fraction was randomly selected per patient, so that all patients were weighted equally and the compared samples remained independent. A schematic overview of the DVH evaluation is shown in Fig. 2. Starting from the original clinically approved adapted plan (GTV, PTV and OAR contours with their DVHs), GTV^AI^ were generated with the proposed workflow, and corresponding PTVs were created using the same margin rules as the original plan (isotropic 3–5 mm GTV expansion).

The plan was then re-optimized to the new target volumes (nnInteractive), with OAR contours unchanged. The resulting dose distribution was evaluated on the original GTV, PTV, and OAR contours, and the new DVHs were compared with the original ones; single-fraction doses were first scaled to the total prescribed course, per standard clinical practice. All plan reoptimizations were performed in the clinical treatment planning system (MRIdian, ViewRay Systems Inc., Oakwood Village, OH, USA) used for the original plans. Dose was computed with the same Monte Carlo algorithm and 2 mm dose-grid size as the clinically approved plan. The delivery technique was IMRT, and the number of beams, segments, the optimization objectives and the constraints were kept identical to those of the corresponding reference plan; only the target volumes (GTV^AI^ and PTV^AI^) were changed. Only the contour-generation step of the workflow is automated (≈ 1 s per fraction), whereas plan reoptimization is a manual step performed in the vendor TPS.

For GTV and PTV, $D_{2\%}$, $D_{98\%}$, and mean dose ($D_{\text{mean}}$) were evaluated (Wilcoxon signed-rank test). Additional violations of GTV $D_{100\%}$ and PTV $D_{95\%}$ were also identified, defined as cases meeting the prescribed dose in the original plan but not in the re-optimized plan; these indicate that GTV^AI^ and PTV^AI^ differ from the originals, so the re-optimized plan may not cover the original volumes identically. For OARs, agreement with the clinical dose limits of Puckett et al. [28] was evaluated, identifying additional violations (limits met in the original plan but exceeded in the re-optimized plan).

## 3 Results

In validation, nnInteractive outperformed MedSAM2: refining affine- and TM-propagated masks improved median DSC by approximately 7% (0.74 to 0.79) and 5% (0.78 to 0.82), respectively; all reported metric values are medians (supplementary material, Table S.2). Given its superior validation performance, the testing phase focused exclusively on nnInteractive.

In both testing datasets, refinement using the nnInteractive model improved mask propagation accuracy in almost all evaluated metrics, reported as median and interquartile range (IQR) (Table 1). For example, in the internal testing dataset, the median Dice Score increased from 0.73 to 0.80 (approximately 10%) for affine propagation. For TM-based propagation, the median Dice Score increased from 0.82 to 0.83. Comparable results were observed for central and peripheral lesions. Some metrics showed negligible or no improvement, notably HD95 (affine: 4.5 to 4.2 mm; TM: unchanged). Similar trends were observed in the external testing dataset (supplementary material, Fig. S.1–S.6). NnInteractive refinement yielded statistically significant improvements in most evaluated metrics in both testing datasets (Wilcoxon signed-rank test; supplementary material, Table S.3). However, HD95 did not exhibit statistically significant changes in the testing datasets regardless of the propagation method. Similarly, for TM-based propagation, no statistically significant differences were observed for HD50 and Surface Dice.

**Table 1:** Segmentation accuracy in the internal and external testing datasets for all evaluated metrics. All values are medians with the interquartile range (IQR) in parentheses. HD95 and HD50 are given in mm; DSC, sDSC, Precision and Recall are dimensionless. The corresponding *p*-values (Wilcoxon signed-rank test) are given in Supplementary Table S.3.

		Affine-propagation	Affine-propagation + nnInteractive	TM-propagation	TM-propagation + nnInteractive
		Median (IQR)			
*Internal dataset*	DSC	0.73 (0.65, 0.80)	0.80 (0.75, 0.84)	0.82 (0.77, 0.85)	0.83 (0.79, 0.86)
	HD95 [mm]	4.5 (3.4, 6.7)	4.2 (3.4, 6.7)	3.4 (3.0, 4.5)	3.4 (3.0, 4.9)
	HD50 [mm]	2.0 (1.5, 3.0)	1.5 (1.5, 1.5)	1.5 (1.5, 1.5)	1.5 (1.5, 1.5)
	sDSC	0.72 (0.51, 0.85)	0.84 (0.68, 0.91)	0.84 (0.75, 0.92)	0.88 (0.77, 0.93)
	Precision	0.74 (0.66, 0.83)	0.76 (0.69, 0.85)	0.81 (0.75, 0.86)	0.77 (0.72, 0.84)
	Recall	0.73 (0.64, 0.81)	0.85 (0.79, 0.91)	0.84 (0.77, 0.89)	0.91 (0.86, 0.94)

*External dataset*	DSC	0.64 (0.41, 0.75)	0.74 (0.62, 0.82)	0.77 (0.68, 0.82)	0.81 (0.70, 0.85)
	HD95 [mm]	6.22 (3.65, 9.15)	6.0 (4.43, 9.0)	4.72 (3.41, 6.0)	4.67 (3.65, 6.22)
	HD50 [mm]	3.0 (1.63, 4.43)	1.63 (1.63, 3.0)	1.63 (1.63, 2.31)	1.63 (1.63, 2.31)
	sDSC	0.54 (0.33, 0.79)	0.68 (0.51, 0.81)	0.78 (0.66, 0.87)	0.79 (0.66, 0.87)
	Precision	0.67 (0.41, 0.78)	0.70 (0.54, 0.80)	0.81 (0.69, 0.88)	0.79 (0.61, 0.86)
	Recall	0.62 (0.39, 0.74)	0.80 (0.67, 0.87)	0.74 (0.63, 0.82)	0.83 (0.74, 0.89)

**Fig. 3:**
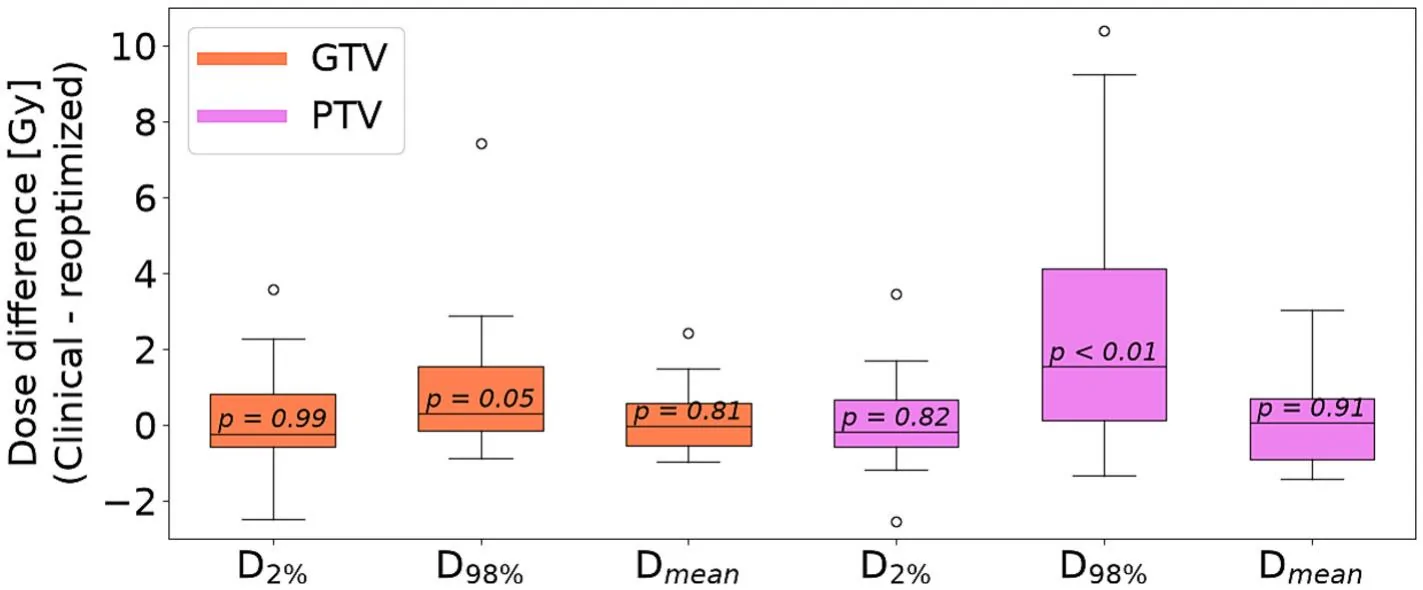
Boxplots of dose differences between clinically approved and reoptimized coverage in selected GTV and PTV DVH metrics ($D_{2\%}$, $D_{98\%}$, and $D_{\text{mean}}$). The presented *p*-values (Wilcoxon signed-rank test) indicate the statistical significance of differences between the compared sets of values.

No statistically significant differences between the reoptimized and the original clinically approved dose distributions were found in GTV DVH parameters; a statistically significant difference was found only in PTV D_98%_ (p<0.01; Wilcoxon signed-rank test, Fig. 3).

**Fig. 4:**
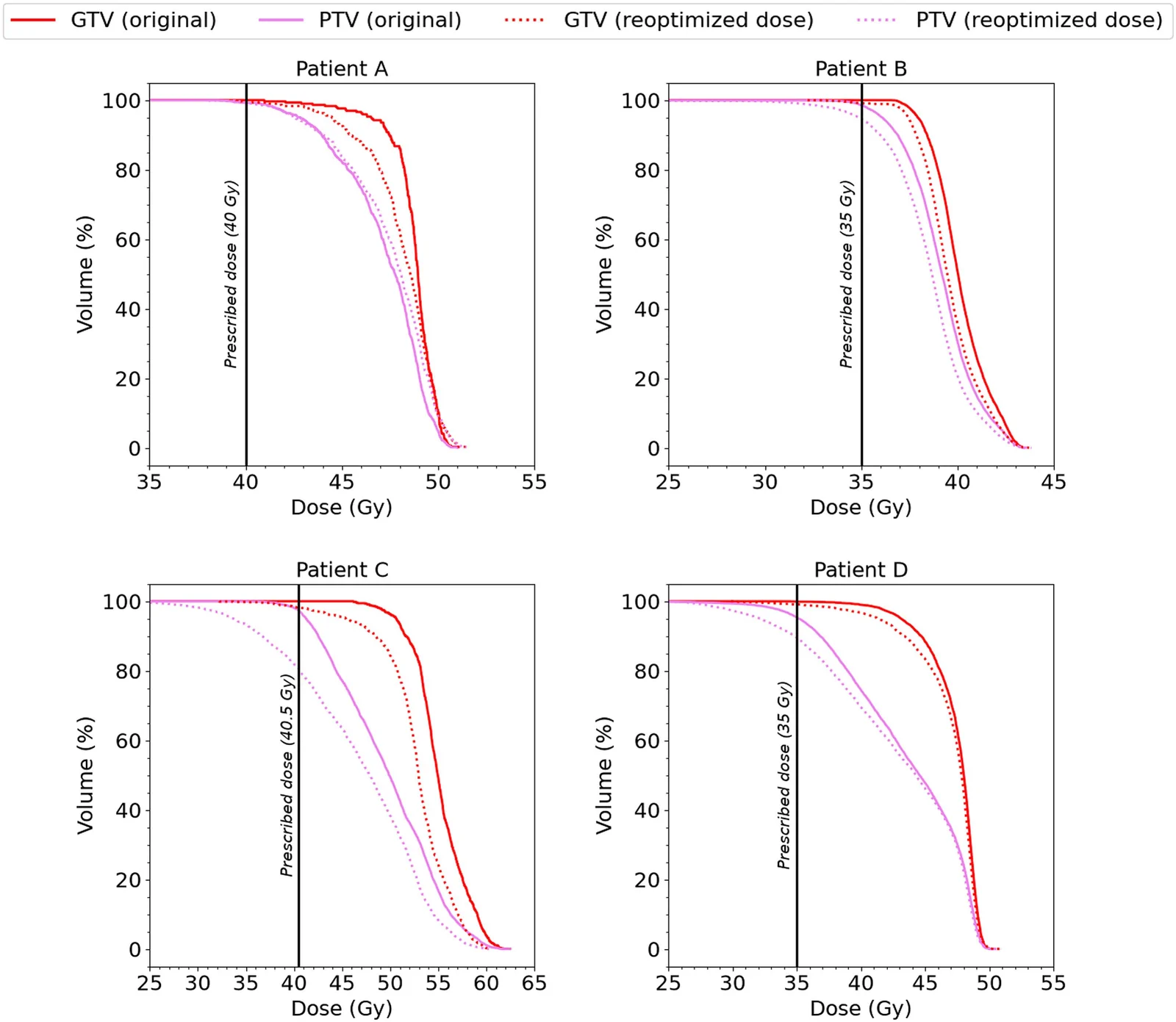
GTV and PTV DVHs for patients with additional violation of GTV $D_{100\%}$ requirement. Full lines indicate original clinically approved DVHs. Dotted lines indicate DVHs for reoptimized dose distributions.

There were 9 additional violations of PTV $D_{95\%}$. In addition, 4 GTV $D_{100\%}$ violations (in 4 of 21 patients) and 3 OAR dose-limit violations (in 2 of 21 patients) were observed (Table 2). The GTV violations all involved peripheral lesions adjacent to the chest wall or abdominal organs (supplementary material, Fig. S.7-S.14), while the OAR violations involved large, irregular central lesions and lung dose constraints (supplementary material, Fig. S.15-S.18). For each violation patient, the supplementary material additionally provides the corresponding dose-volume histograms for the target volumes and organs at risk, including the ipsilateral lung. For the four GTV-violation patients, the reoptimized dose distributions reduced GTV coverage below the prescription compared to the original plans (Fig. 4). Among the nine PTV $D_{95\%}$ violations, seven occurred in peripheral and two in central lesions. The reduction in PTV $D_{95\%}$ relative to the original plan was small in most cases, with a median of 2.4 Gy (6%) and eight of nine reductions below 9%; a single case showed a larger reduction of 7.4 Gy (18%). Notably, in these nine cases the GTV segmentation accuracy was in line with the overall cohort (median Dice 0.82, median HD95 4.2 mm).

**Table 2:** Additional GTV $D_{100\%}$ and OAR dose-limit violations after reoptimization. For GTV $D_{100\%}$, the limit corresponds to the prescribed dose. For OARs, the limits correspond to dose limits published in the study by Puckett et al. [28].

	Patient	$D_{\text{prescribed}}$	Metric	Original	Reoptimized	Prescription/Limit
*GTV*	A	40 Gy / 5 fr.	GTV $D_{100\%}$	42.1 Gy	39.8 Gy	40 Gy
	B	35 Gy / 5 fr.	GTV $D_{100\%}$	37.0 Gy	34.3 Gy	35 Gy
	C	40.5 Gy / 3 fr.	GTV $D_{100\%}$	46.6 Gy	37.5 Gy	40.5 Gy
	D	35 Gy / 5 fr.	GTV $D_{100\%}$	38.5 Gy	33.0 Gy	35 Gy

*OAR*	E	50 Gy / 10 fr.	Ipsi. lung V_16Gy_	25.6%	31.0%	30%
	E	50 Gy / 10 fr.	Ipsi. lung $D_{\mathrm{mean}}$	11.0 Gy	12.3 Gy	12.0 Gy
	F	50 Gy / 10 fr.	Ipsi. lung V_16Gy_	28.4%	31.4%	30%

## 4 Discussion

The validation showed that foundation-model refinement of affine- or TM-propagated GTV masks was feasible and improved segmentation accuracy, with nnInteractive outperforming MedSAM2 on most metrics (Table S.2); MedSAM2 was superior only in Precision. Precision was also assessed in the testing datasets (Table 1), where nnInteractive refinement produced Precision values comparable to those of the baseline propagation methods. The superior performance of nnInteractive was expected, as it is designed for 3D medical segmentation, whereas MedSAM2's temporal-propagation architecture was less suited to this task, particularly given the typical MRI slice thickness (∼3 mm). Blöcker et al. [25] similarly identified nnInteractive as more suitable for GTV segmentation.

In both testing datasets, nnInteractive refinement produced statistically significant improvements in segmentation metrics compared with affine- or TM-based propagation (Table 1). The improvement was most pronounced for affine propagation (up to 15% in Dice Score), whereas gains over TM-based propagation were smaller, since TM (i.e., deformable image registration) already yielded inherently more conformal contours; TM with refinement therefore gave the best accuracy, and affine with refinement the largest gain at lower computational cost. The strong performance of TM-based propagation observed here is in line with Wei et al. [15], who reported that TransMorph outperformed conventional registration and auto-segmentation approaches for contour propagation in MRI-guided radiotherapy of lung cancer. Only marginal improvements were observed for HD95, which is sensitive to large deviations rather than the minor local corrections that dominated the refinements; such large deviations were rare in the baseline propagations. This indicated that the foundation model was precise in bulk-volume segmentation but less effective at correcting major deviations. Importantly, even where differences did not reach statistical significance, the refinement may still be clinically useful: it delivered consistent, fully automated contour adaptation in approximately one second, reducing manual workload without degrading segmentation quality, which is relevant considering the substantial share of on-couch time that contour adaptation takes [5], [6].

The DVH evaluation confirmed that the proposed workflow did not introduce a systematic negative impact on the plan quality of adapted treatment plans. Median deviations between clinically approved and reoptimized values were close to zero, and no differences reached statistical significance (Fig. 3); the only exception was PTV $D_{98\%}$, for which a decrease in coverage was observed. The emphasis on evaluating the dose-volume consequences of automated contour adaptation, rather than segmentation accuracy alone, was in agreement with Smolders et al. [27], who identified the dose-volume assessment of propagated contours as a crucial step toward clinical implementation. This aligned with our own observations: GTV segmentation accuracy in the PTV $D_{95\%}$-violation cases was acceptable, yet these cases exhibited additional PTV coverage violations. Consistently, several studies have reported that geometric agreement metrics correlate only weakly with the dosimetric consequences of contour differences [29], [30], reinforcing the need to complement geometric evaluation with a dose-volume analysis such as the one performed here.

Nevertheless, although most metrics did not show significant degradation, several outliers with unacceptable coverage decrease were identified. As presented in Results and Table 2, two of the four additional GTV $D_{100\%}$ violations (Patients A and B) were minor (0.2 and 0.7 Gy below the prescription), while the other two (Patients C and D) were more considerable (2–3 Gy below the prescription). Even though some of these violations are numerically small, they remain failures at the fraction level if the original contours are adopted as reference.

The 9 additional PTV $D_{95\%}$ violations were expected, since plans are typically optimized to this or similar DVH metrics, so any change in the optimization GTV can noticeably change PTV coverage. The PTV is a central construct in treatment planning, and adequate PTV coverage remains an important objective, even though it is not itself an anatomical structure. Importantly, these did not represent reduced coverage of the adapted PTV in the reoptimized plan. Instead, they reflected fraction-level reductions in coverage of the original clinically approved PTV when the reoptimized dose distribution is applied, and their clinical relevance over a full treatment course remains uncertain.

Only 3 additional OAR violations occurred, all in the ipsilateral lung (V_16Gy_ exceeded by approximately 1% in two cases; $D_{\text{mean}}$ by 0.3 Gy in one) and likely mitigable by minor plan adjustments. For these two patients (E and F), Precision was low (0.63 and 0.41) while Recall remained high (0.79 and 0.93), against a cohort median Precision of 0.77. This pattern indicated over-segmentation: the adapted target volume (GTV^AI^) had exceeded the original contour, which drove additional dose into the adjacent lung and thereby explained these OAR violations. All GTV $D_{100\%}$ violations involved peripheral lesions adjacent to the chest wall or abdominal organs, whereas OAR violations involved central, irregular lesions. Clinical implementation should therefore include a QA strategy for peripheral lesions and increased caution for central, irregular ones, which were segmented less precisely; integrating dosimetrically informed contour editing [31] is another option for preventing DVH constraint violations in the adapted plan.

The main limitation of this manuscript is the lack of external DVH evaluation. Due to the unavailability of the optimization objectives for the adapted treatment plans in the external dataset, such evaluation could not be practically performed. A further limitation is the restricted scope of the dose-volume evaluation, which was performed on the internal cohort only and on a single randomly selected fraction per patient (*n* = 21). One fraction per patient was drawn at random so that patients with more fractions did not dominate the comparison and so that the paired samples remained independent. Plan reoptimization was a manual step in the clinical treatment planning system, which offers no scripting interface, so all optimization objectives, constraints and beam settings had to be re-entered for every plan; reoptimizing all 96 fractions from the internal testing dataset was therefore not achievable. With 21 fractions, the analysis was powered to detect only large systematic effects. The dose-volume results should therefore be regarded as hypothesis-generating and require confirmation in a larger, ideally multi-institutional cohort. Evaluating the accumulated dose over the full treatment course, as recently done for deep learning-based OAR segmentation in 0.35 T MR-guided lung radiotherapy by Xiong et al. [32], would provide a more complete picture and represents an important direction for future work. In addition, clinical-acceptability scoring and inter-observer evaluation of the adapted contours were not performed in this retrospective feasibility study; such assessments require multiple independent expert raters and are planned in a dedicated follow-up study. Combined geometric, dose-volume and clinical-acceptance evaluation of automatically delineated structures, as performed for head and neck MR-Linac patients by Koteva et al. [33], provides a template for such an assessment.

This study presented a foundation-model workflow for GTV contour adaptation in online adaptive MRI-Linac radiotherapy. Combined with advanced OAR contour propagation such as TransMorph [15], it could enable a fully automated daily adaptation workflow. Comparable approaches are also being explored for other tumor sites [34], [35]. In conclusion, daily refinement with nnInteractive allows for automated GTV contour adaptation workflow with superior accuracy while maintaining plan quality in the majority of patients.

## CRediT authorship contribution statement

**Denis Dudas:** Writing – original draft, Visualization, Validation, Software, Methodology, Formal analysis, Data curation, Conceptualization. **Tom Julius Blöcker:** Writing – review & editing, Software. **Miguel A. Palacios:** Writing – review & editing. **Chengtao Wei:** Writing – review & editing. **Chukwuka Eze:** Writing – review & editing. **Stefanie Corradini:** Writing – review & editing. **Claus Belka:** Writing – review & editing. **Christopher Kurz:** Writing – review & editing, Methodology, Conceptualization. **Guillaume Landry:** Writing – review & editing, Project administration, Funding acquisition, Conceptualization.

## Funding

This work was supported by the Bavarian Cancer Research Center (BZKF) Lighthouse - Image Guidance in Local Therapies.

## Declaration of competing interest

The authors declare that they have no known competing financial interests or personal relationships that could have appeared to influence the work reported in this paper.
