# Supplementary material for: Automated workflow for target volume propagation in online adaptive magnetic resonance guided radiotherapy for lung lesions using foundation models
Source: Phys Imaging Radiat Oncol. 2026 Sep 7;41:101077. doi: 10.1016/j.phro.2026.101077 (PMC13580049; doi:10.1016/j.phro.2026.101077)
Supplement: Supplementary file 1 [file mmc1.pdf]

Supplementary material

Fractionation regimen	Number of patients
40.5 Gy/ 3 fr.	6
45 Gy/ 3 fr.	3
30 Gy/ 5 fr.	1
35 Gy/ 5 fr.	2
40 Gy/ 5 fr.	1
60 Gy/ 8 fr.	2
40 Gy/ 10 fr.	1
45 Gy/ 10 fr.	1
50 Gy/ 10 fr.	3

Table S.1 Fractionation regimens by patients in the internal testing dataset.

	DSC	HD95 [mm]	HD50 [mm]	sDSC	Precision	Recall
Baseline prop.			Median (IQR)			
Affine prop.	0.74 (0.61, 0.83)	4.2 (3.4, 6.0)	1.5 (1.5, 3.0)	0.74 (0.57, 0.88)	0.76 (0.64, 0.83)	0.73 (0.61, 0.84)
TransMorph prop.	0.78 (0.72, 0.83)	3.4 (3.2, 4.3)	1.5 (1.5, 1.5)	0.83 (0.75, 0.9)	0.82 (0.72, 0.87)	0.76 (0.7, 0.82)
nnInteractive refinement						
Affine prop. + nnInteractive	**0.79 (0.72, 0.84)**	**4.2 (3.6, 5.3)**	**1.5 (1.5, 1.5)**	**0.85 (0.74, 0.92)**	0.78 (0.66, 0.88)	**0.83 (0.77, 0.87)**
TransMorph prop. + nnInteractive	**0.82 (0.74, 0.86)**	**3.7 (3.0, 5.4)**	**1.5 (1.5, 1.5)**	**0.85 (0.74, 0.92)**	0.78 (0.69, 0.87)	**0.87 (0.81, 0.90)**
MedSAM2 refinement						
Affine prop. + MedSAM2	0.76 (0.68, 0.8)	6.0 (4.8, 8.2)	1.5 (1.5, 1.6)	0.75 (0.68, 0.82)	**0.88 (0.81, 0.92)**	0.70 (0.58, 0.75)
TransMorph prop. + MedSAM2	0.75 (0.69, 0.8)	5.8 (4.2, 6.9)	1.5 (1.5, 2.1)	0.76 (0.69, 0.84)	**0.89 (0.78, 0.94)**	0.69 (0.61, 0.77)

Table S.2: Validation segmentation metrics (median, IQR) for the best prompting strategy, based on three orthogonal masks intersecting at the center of the tumor volume. Bold font outlines the superior refinement method (for a given metric) compared to the baseline.

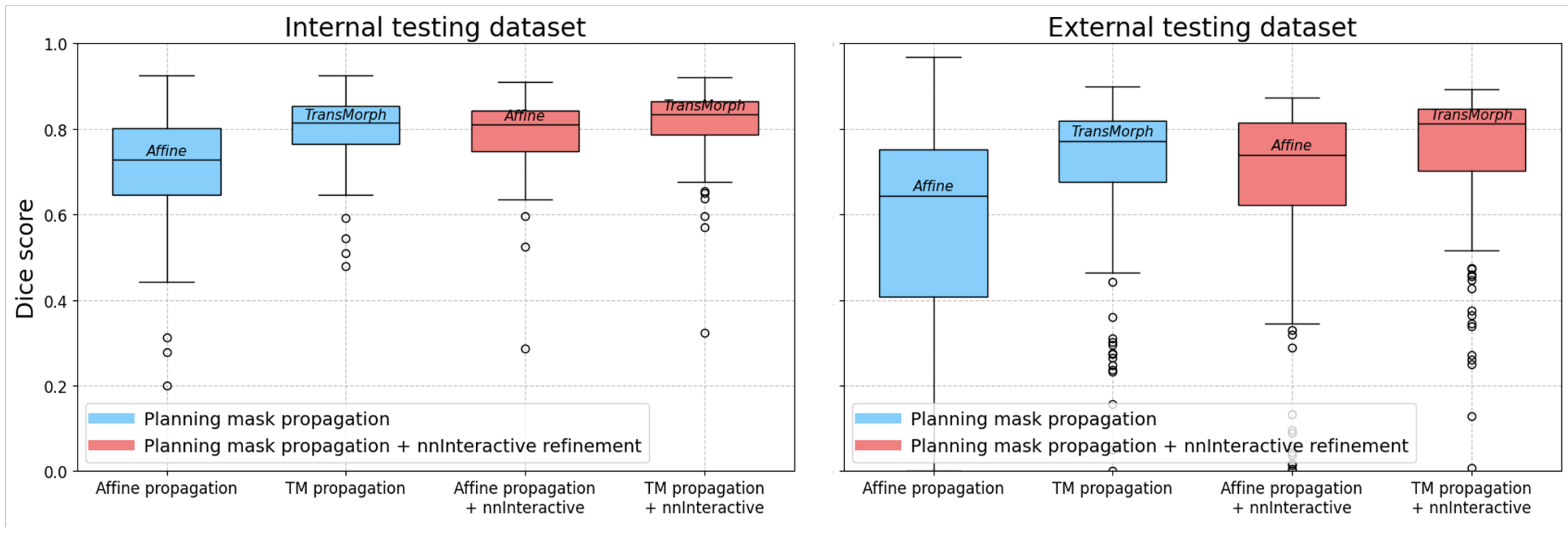

Figure S.1 Boxplots of DSC in internal and external datasets. Whiskers indicate interval (Q1-1.5*IQR, Q3+1.5*IQR) and outliers, which are not within this interval, are displayed as individual points.

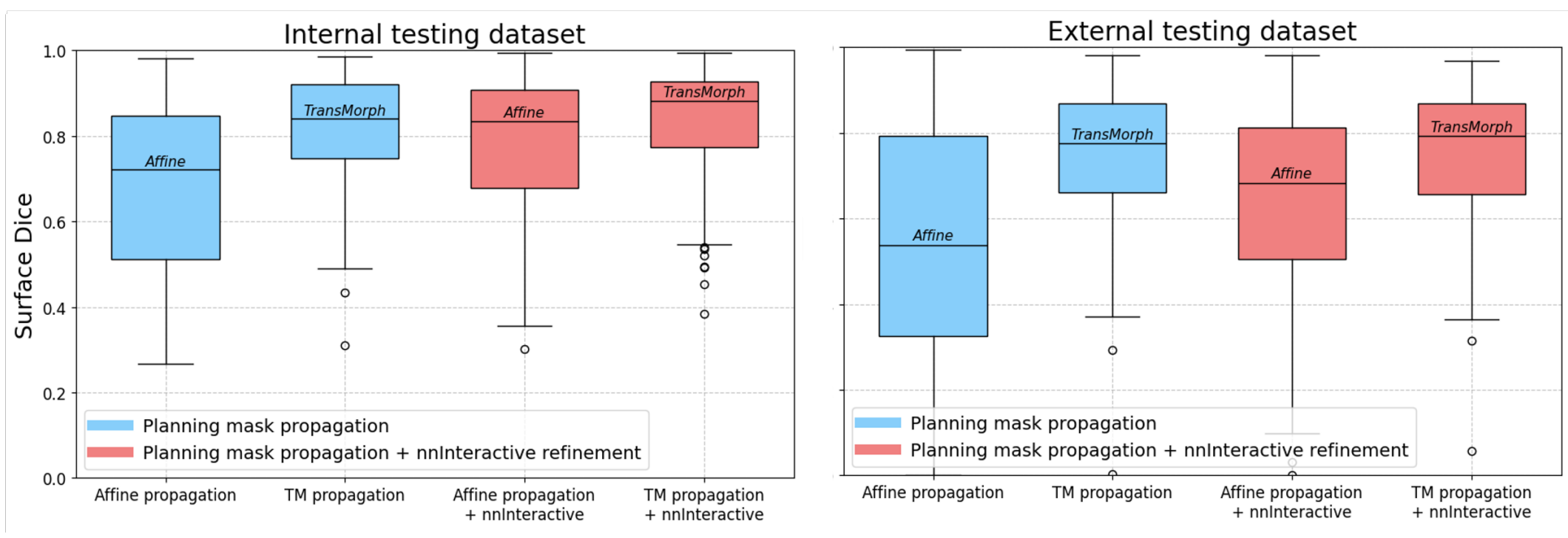

Figure S.2 Boxplots of sDSC in internal and external datasets. Whiskers indicate interval (Q1-1.5*IQR, Q3+1.5*IQR) and outliers, which are not within this interval, are displayed as individual points.

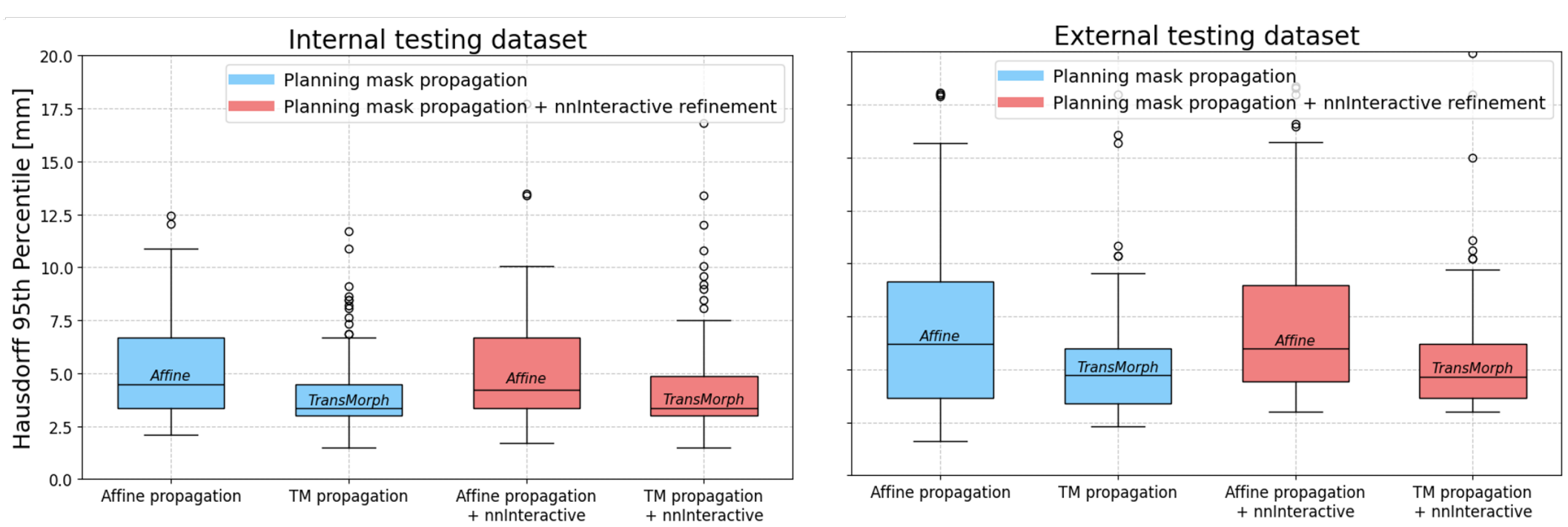

Figure S.3 Boxplots of HD95 in internal and external datasets. Whiskers indicate interval (Q1-1.5*IQR, Q3+1.5*IQR) and outliers, which are not within this interval, are displayed as individual points.

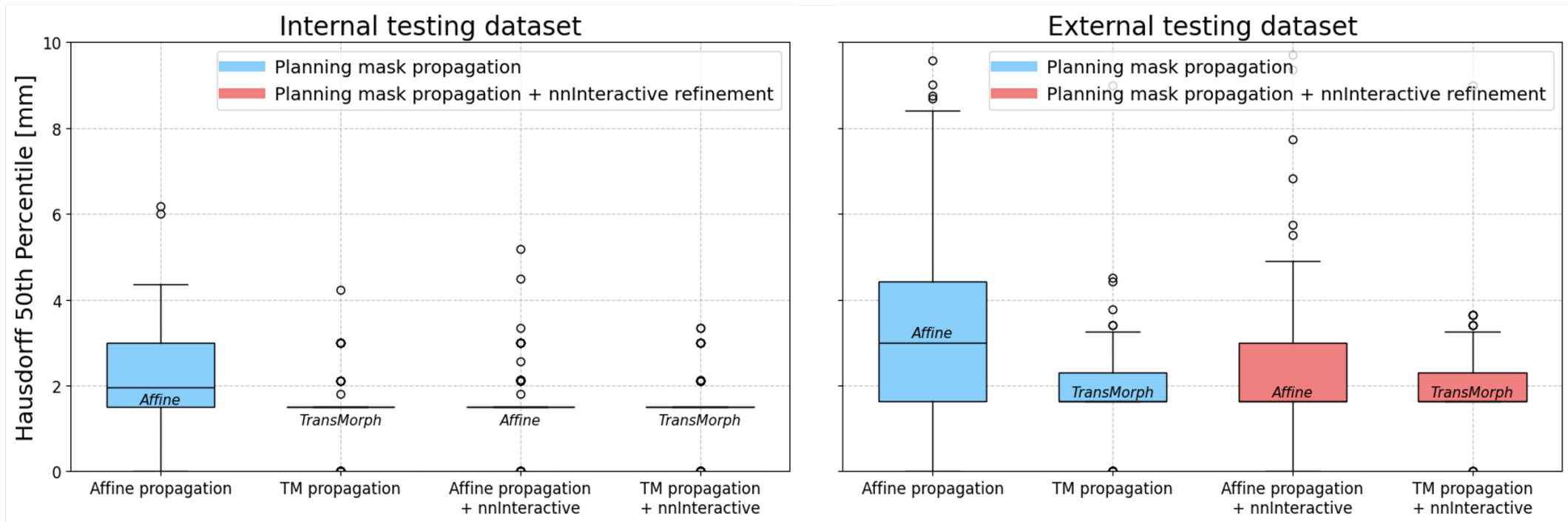

Figure S.4 Boxplots of HD50 in internal and external datasets. Whiskers indicate interval (Q1-1.5*IQR, Q3+1.5*IQR) and outliers, which are not within this interval, are displayed as individual points.

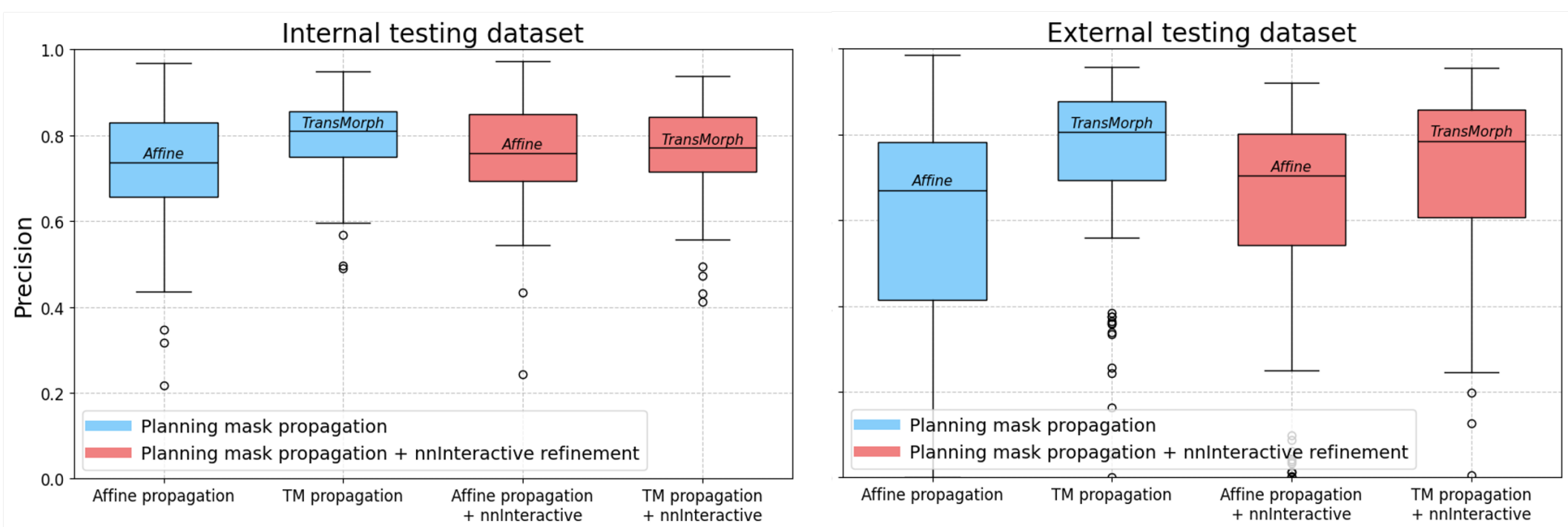

Figure S.5 Boxplots of Precision in internal and external datasets. Whiskers indicate interval (Q1-1.5*IQR, Q3+1.5*IQR) and outliers, which are not within this interval, are displayed as individual points.

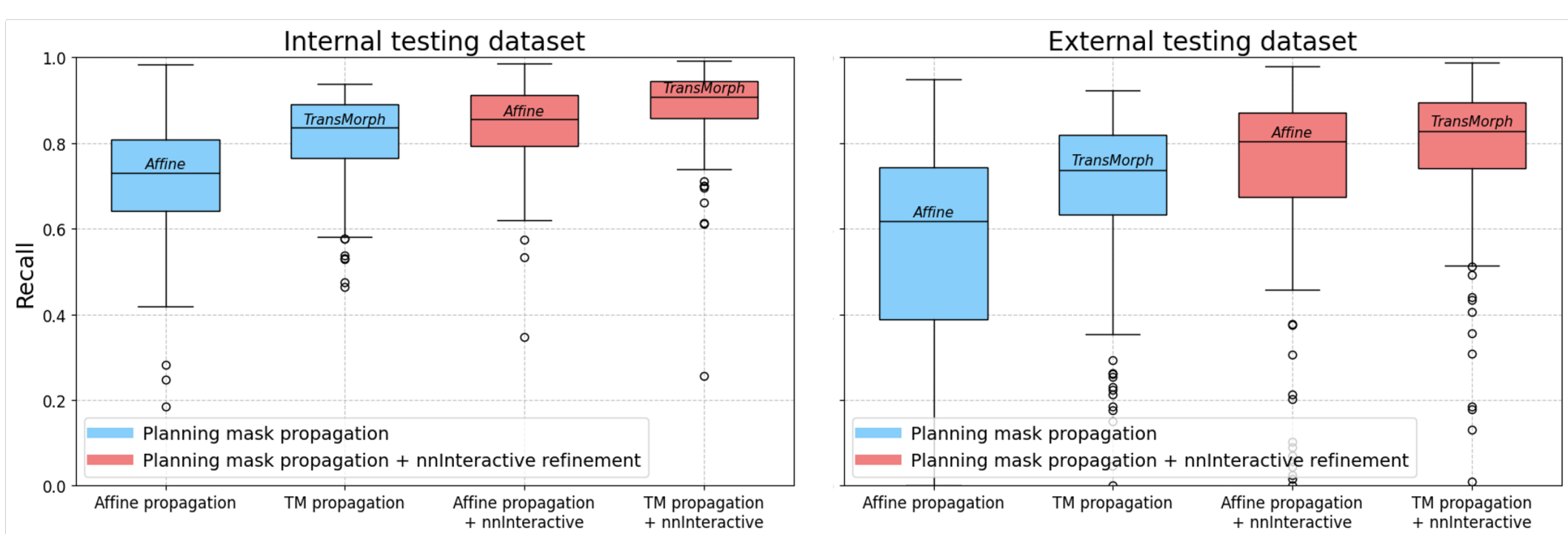

Figure S.6 Boxplots of Recall in internal and external datasets. Whiskers indicate interval (Q1-1.5*IQR, Q3+1.5*IQR) and outliers, which are not within this interval, are displayed as individual points.

Table S.3: Wilcoxon signed-rank test p-values, by segmentation metrics, for internal and external testing datasets. Compared are Affine and TM baseline results with nnInteractive-based refinements. The corresponding metric values (median, IQR) are given in Table 1 of the main manuscript.

	Internal testing		*External testing*	
	Affine	TransMorph	Affine	TransMorph
Dice	**<0.001**	**<0.001**	**<0.001**	**<0.001**
HD95 [mm]	0.42	0.48	0.95	0.51
HD50 [mm]	**<0.001**	0.22	**<0.001**	0.99
SurfDice	**<0.001**	0.17	**<0.001**	0.11
Precision	**<0.001**	**0.004**	**<0.001**	**0.004**
Recall	**<0.001**	**<0.001**	**<0.001**	**<0.001**

Additional GTV violations

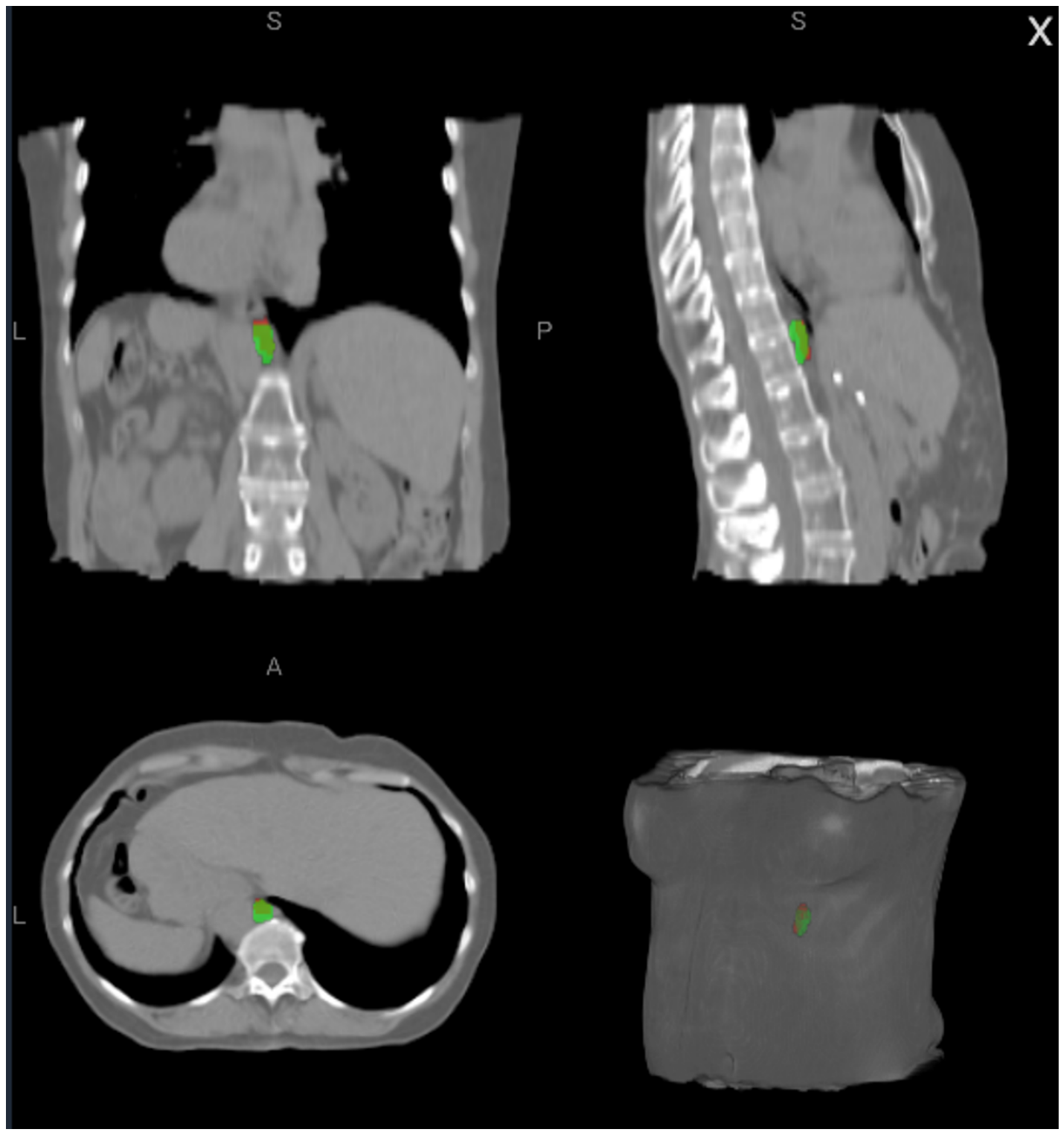

Figure S.7 Additional violation of GTV D_{100} - Patient A. $D_{\text{prescribed}}$ = 40 Gy/ 5 fr., GTV D100 (original/reoptimized) = 42.1 Gy/ 39.8 Gy. The red contour indicates the original clinically approved GTV; the green contour indicates the GTV generated by the proposed workflow (GTV^{AI}).

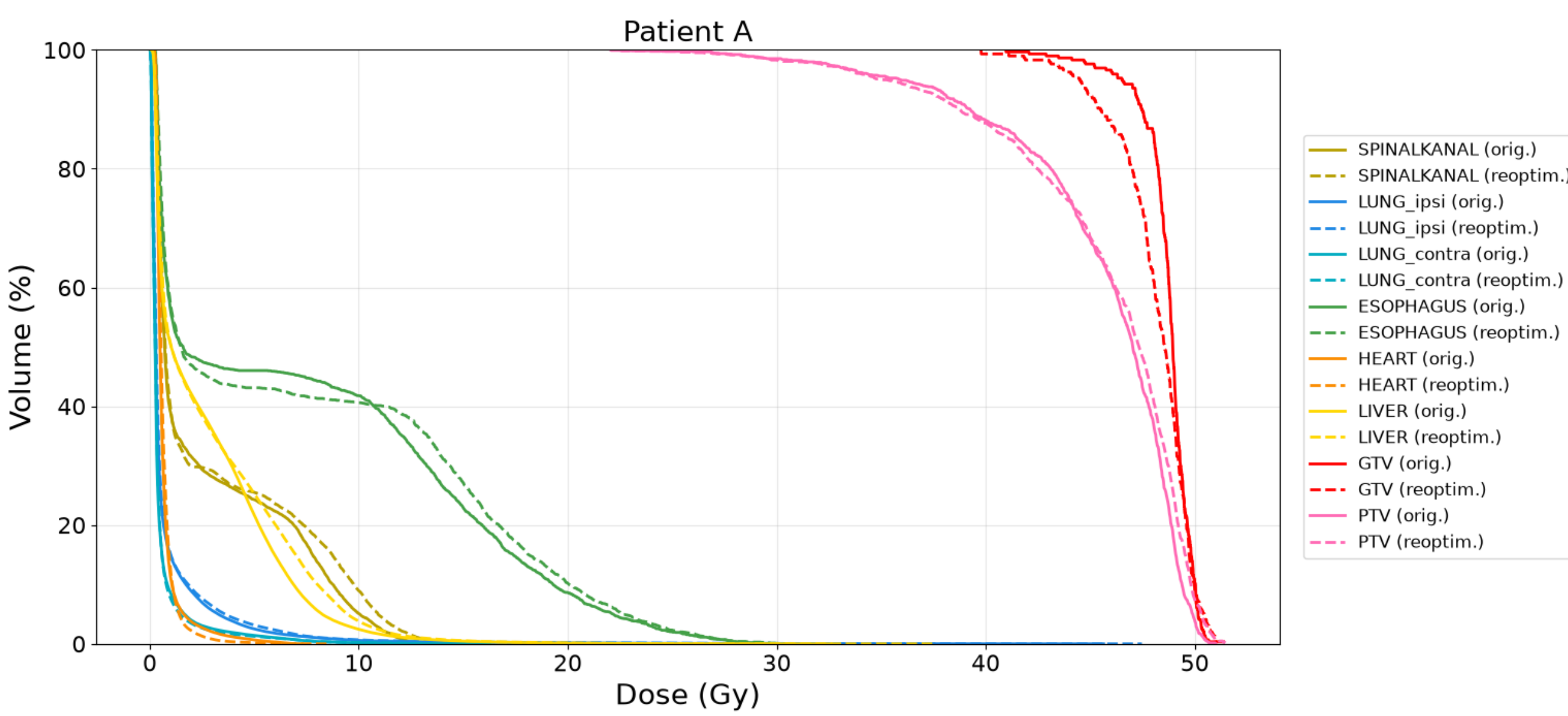

Figure S.8 Dose-volume histograms for Patient A. Solid lines indicate the original clinically approved plan; dashed lines indicate the reoptimized plan (optimized to the workflow-generated GTV^{AI}). Curves are shown for the target volumes and the organs at risk, including the ipsilateral lung.

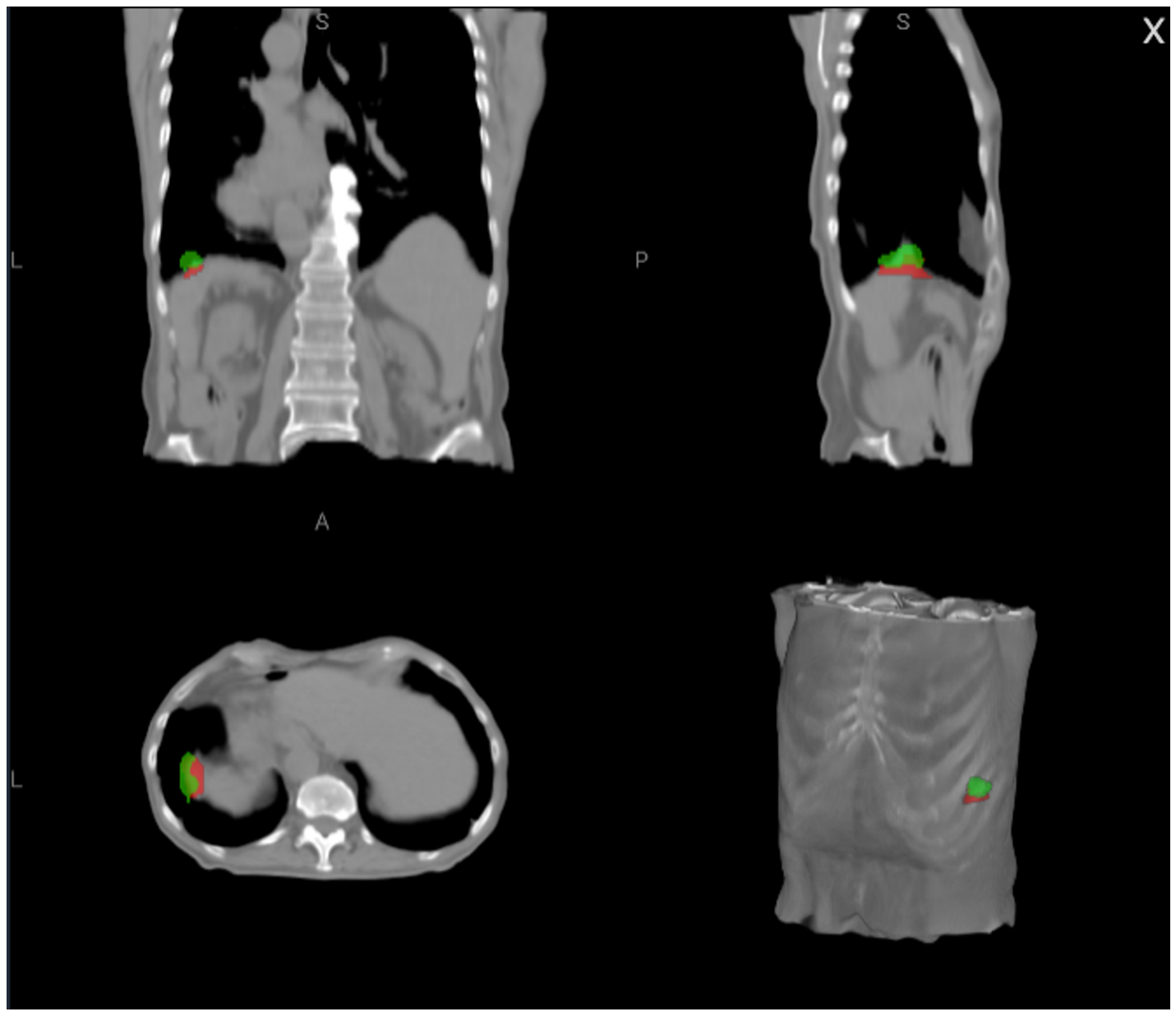

Figure S.9 Additional violation of GTV D_{100} - Patient B. $D_{\text{prescribed}}$ = 35 Gy/5 fr., GTV D100 (original/reoptimized) = 37.0 Gy/ 34.3 Gy. The red contour indicates the original clinically approved GTV; the green contour indicates the GTV generated by the proposed workflow (GTV^{AI}).

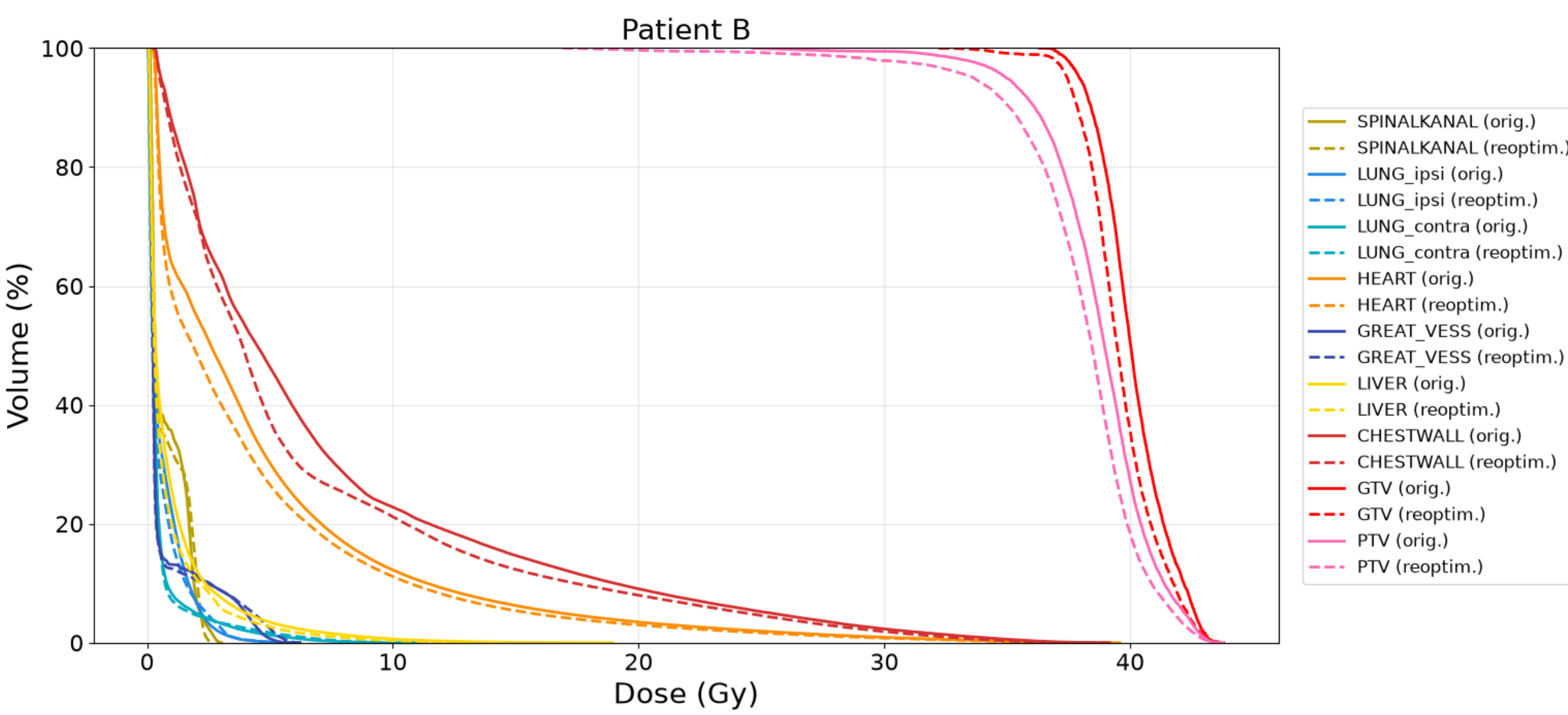

Figure S.10 Dose-volume histograms for Patient B. Solid lines indicate the original clinically approved plan; dashed lines indicate the reoptimized plan (optimized to the workflow-generated GTV^{AI}). Curves are shown for the target volumes and the organs at risk, including the ipsilateral lung.

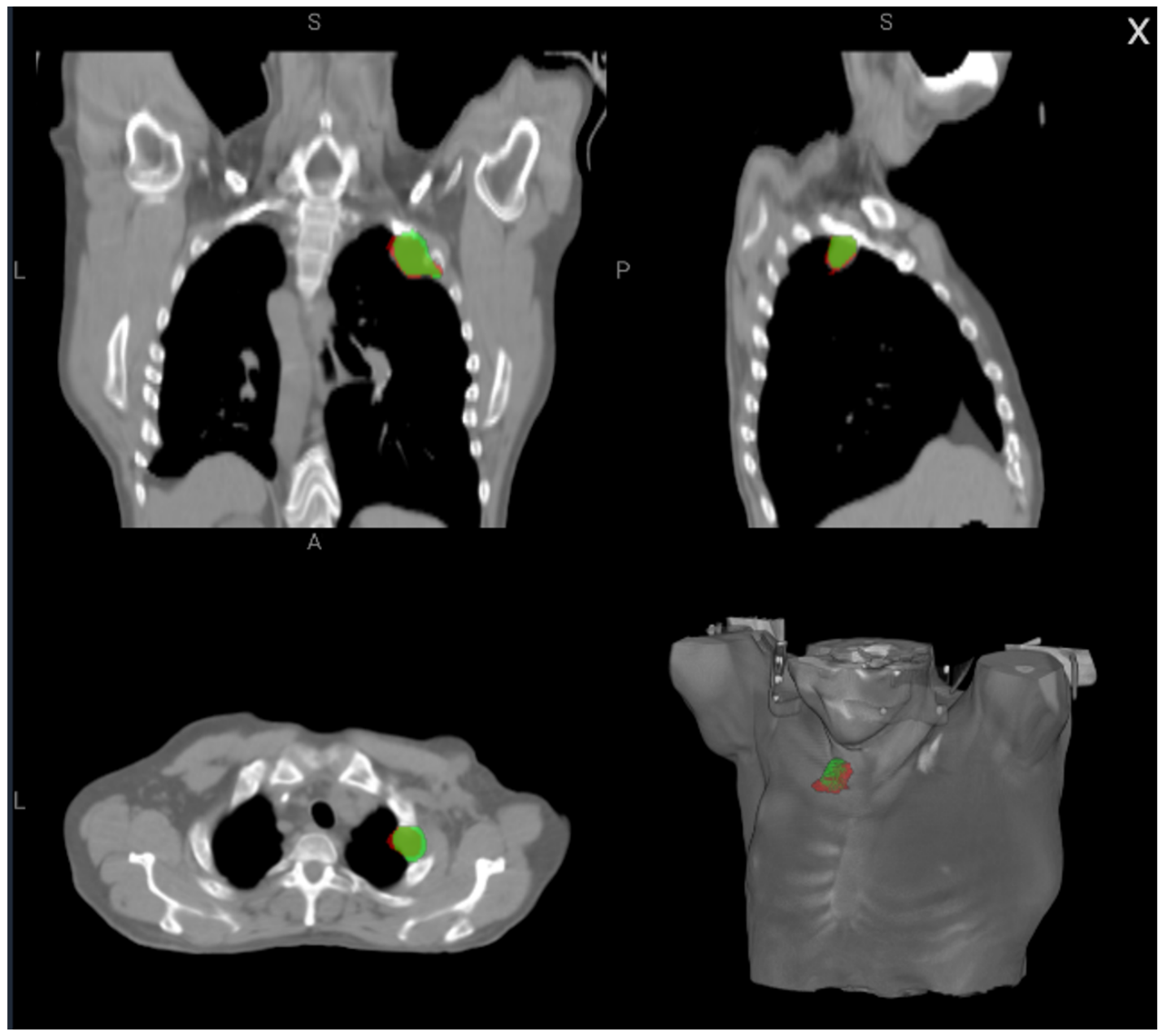

Figure S.11 Additional violation of GTV D_{100} - Patient C. $D_{\text{prescribed}}$ = 40.5 Gy/3 fr., GTV D100 (original/reoptimized) = 46.6 Gy/ 37.5 Gy. The red contour indicates the original clinically approved GTV; the green contour indicates the GTV generated by the proposed workflow (GTV^{AI}).

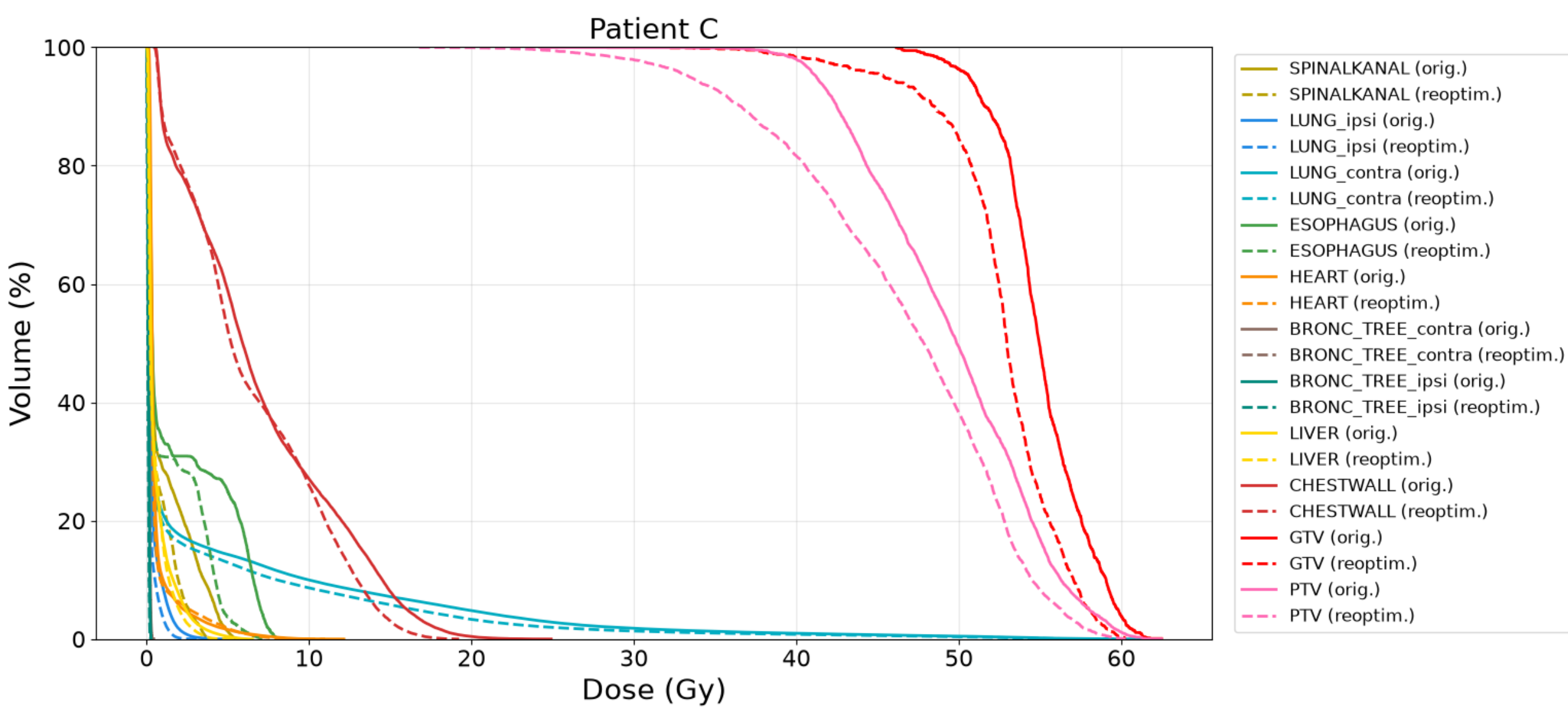

Figure S.12 Dose-volume histograms for Patient C. Solid lines indicate the original clinically approved plan; dashed lines indicate the reoptimized plan (optimized to the workflow-generated GTV^{AI}). Curves are shown for the target volumes and the organs at risk, including the ipsilateral lung.

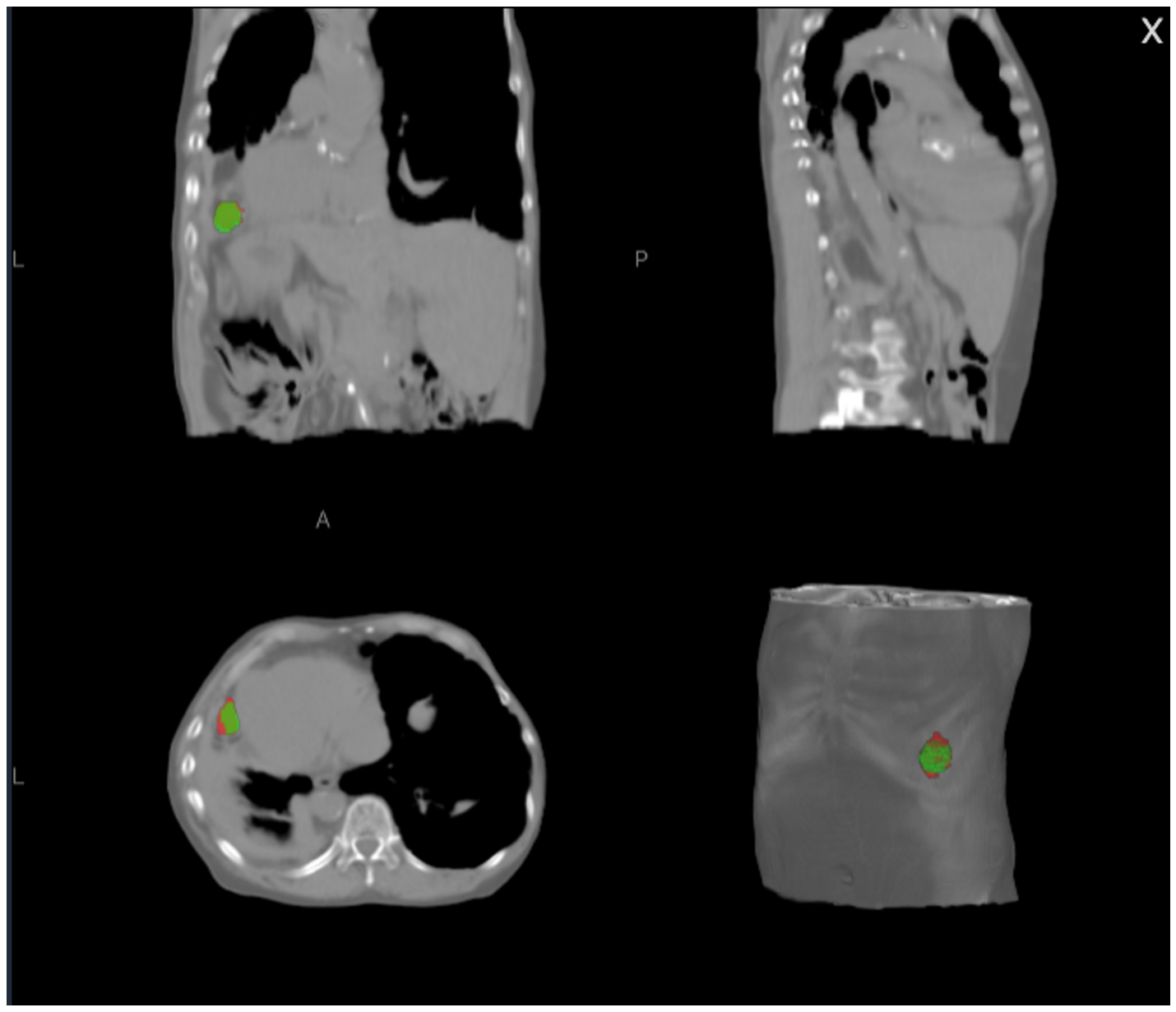

Figure S.13 Additional violation of GTV D_{100} - Patient D. $D_{\text{prescribed}} = 35$ Gy/5 fr., GTV D100 (original/reoptimized) = 38.5 Gy/ 33.0 Gy. The red contour indicates the original clinically approved GTV; the green contour indicates the GTV generated by the proposed workflow (GTV^{AI}).

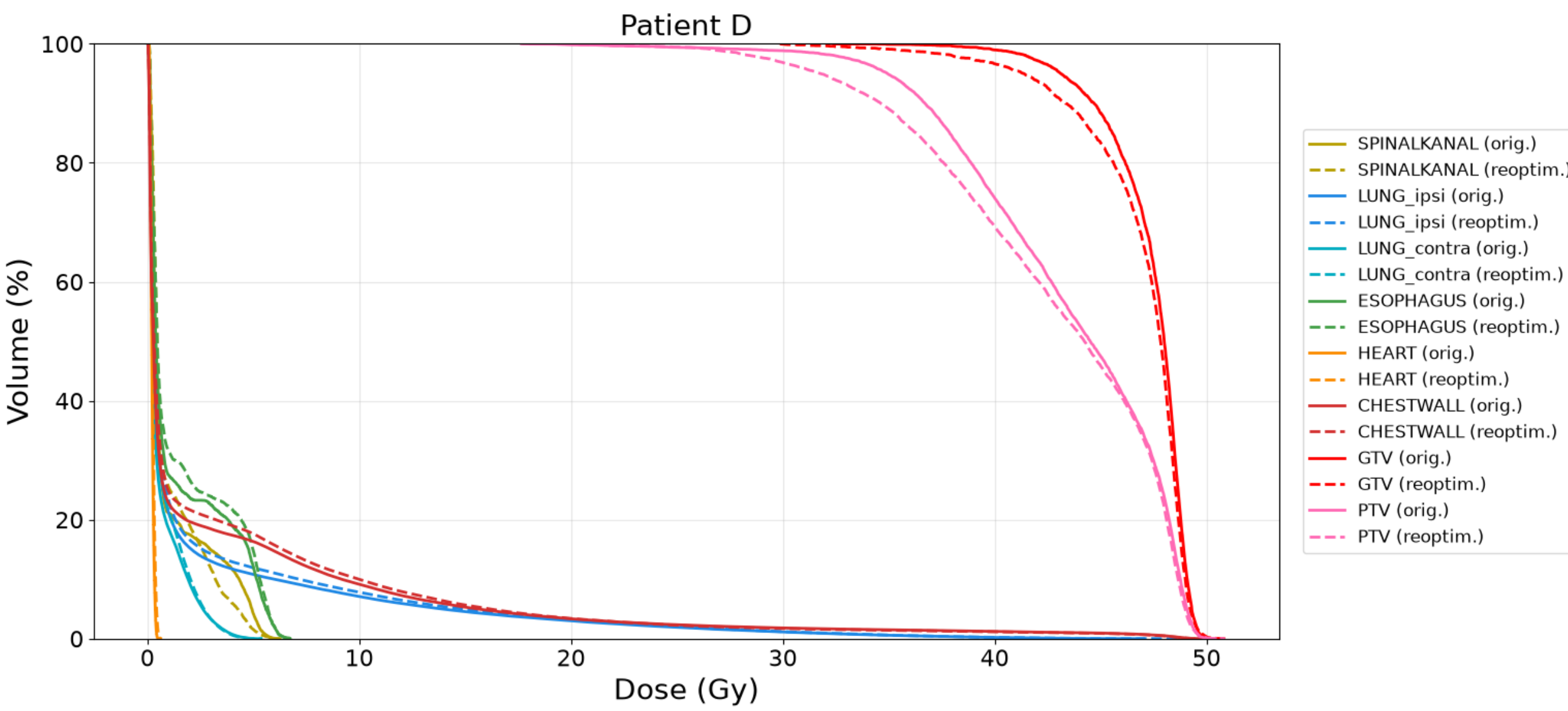

Figure S.14 Dose-volume histograms for Patient D. Solid lines indicate the original clinically approved plan; dashed lines indicate the reoptimized plan (optimized to the workflow-generated GTV^{AI}). Curves are shown for the target volumes and the organs at risk, including the ipsilateral lung.

Additional OAR violations

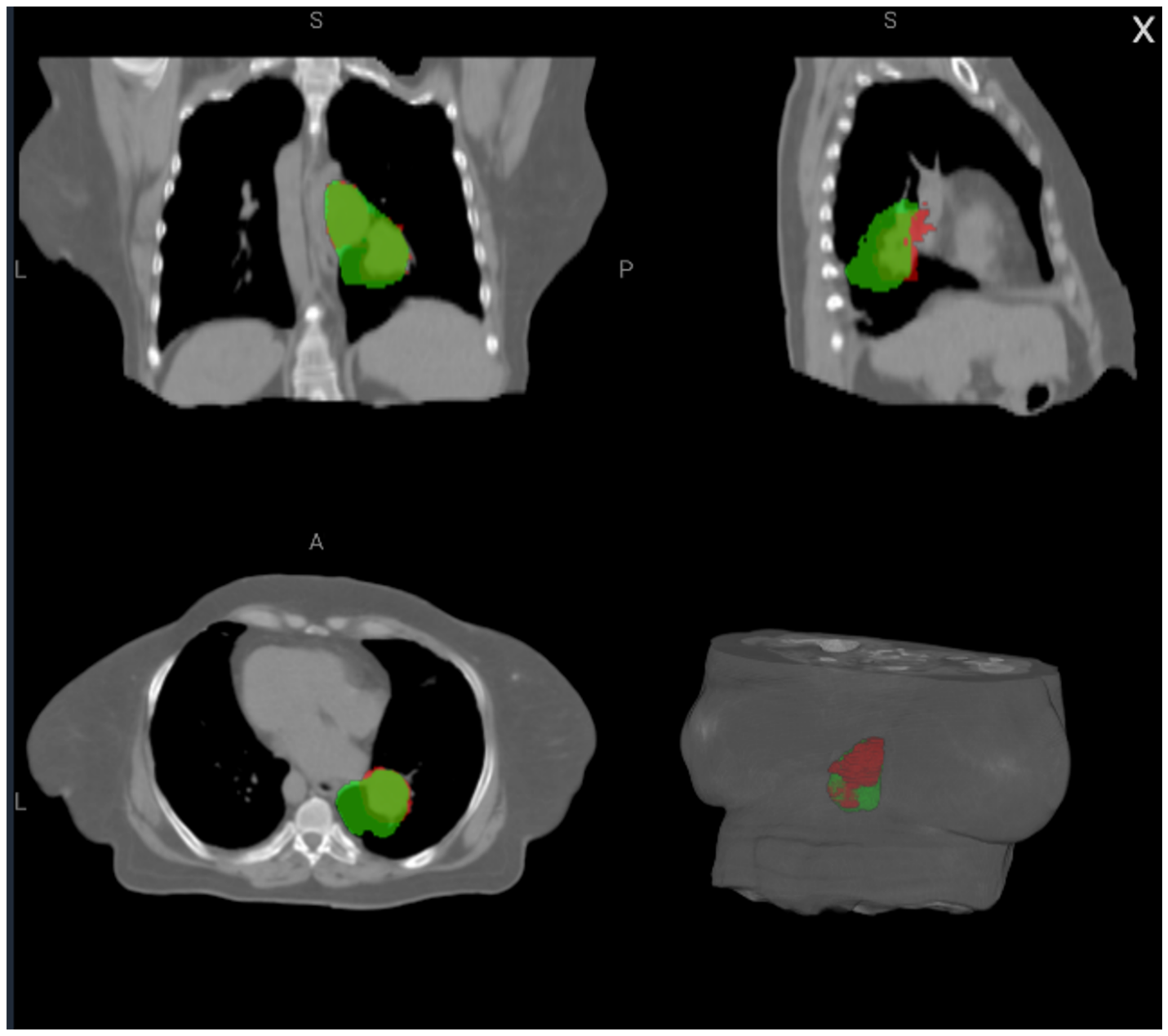

Figure S.15 Additional violation of Ipsilateral lung V16 and D_{mean} - Patient E. $D_{\text{prescribed}} = 50$ Gy/10 fr., V16 (original/reoptimized) = 25.6%/ 31.0% (limit=30%), D_{mean} (original/reoptimized) = 11.0 Gy/ 12.3 Gy (limit=12.0 Gy). The red contour indicates the original clinically approved GTV; the green contour indicates the GTV generated by the proposed workflow (GTV^{AI}).

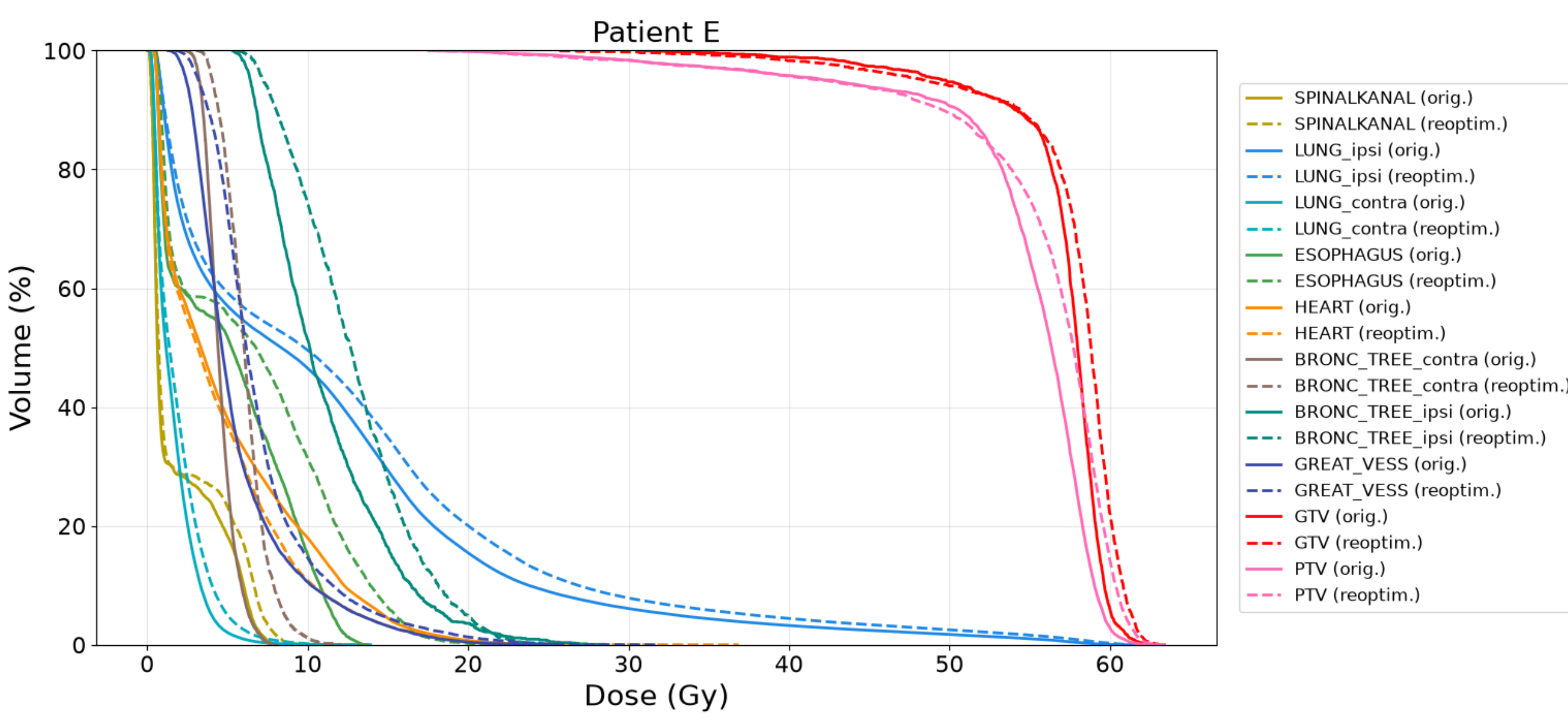

Figure S.16 Dose-volume histograms for Patient E. Solid lines indicate the original clinically approved plan; dashed lines indicate the reoptimized plan (optimized to the workflow-generated GTV^{AI}). Curves are shown for the target volumes and the organs at risk, including the ipsilateral lung.

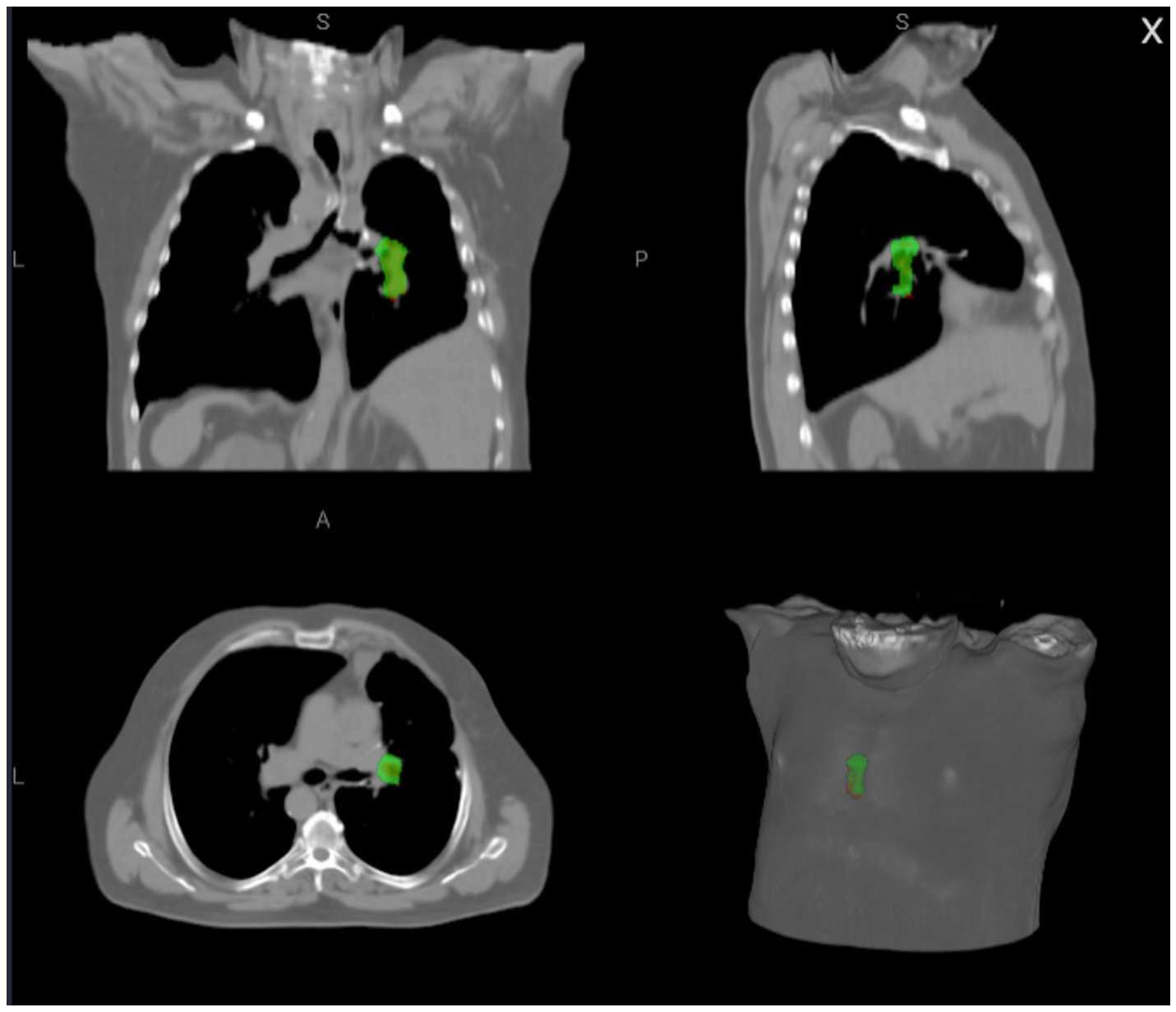

Figure S.17 Additional violation of Ipsilateral lung V16 and D_{mean} - Patient F. $D_{\text{prescribed}}$ = 50 Gy/10 fr., V16 (original/reoptimized) = 28.4%/ 31.4% (limit=30%). The red contour indicates the original clinically approved GTV; the green contour indicates the GTV generated by the proposed workflow (GTV^{AI}).

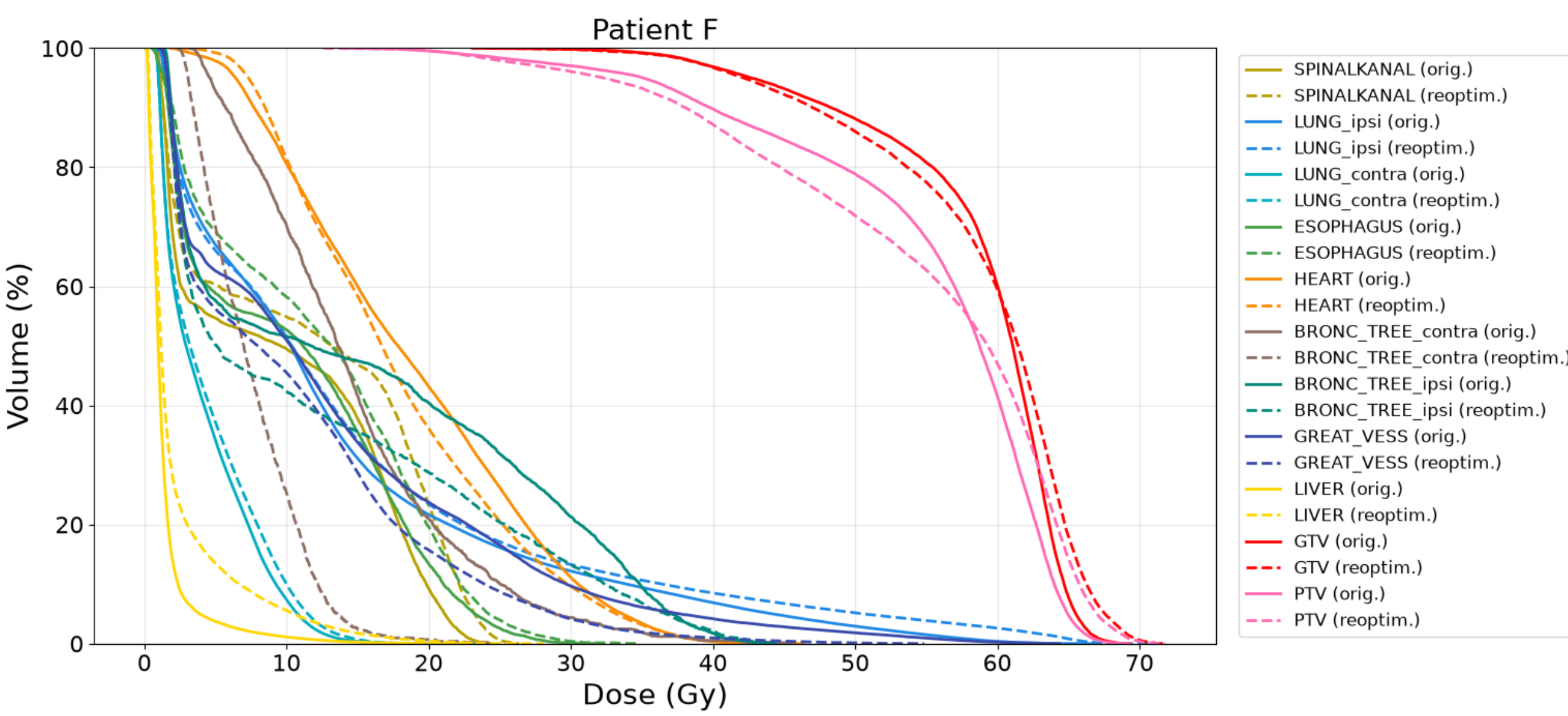

Figure S.18 Dose-volume histograms for Patient F. Solid lines indicate the original clinically approved plan; dashed lines indicate the reoptimized plan (optimized to the workflow-generated GTV^{AI}). Curves are shown for the target volumes and the organs at risk, including the ipsilateral lung.
